# The PB1 protein of influenza A virus inhibits the innate immune response by targeting MAVS for NBR1-mediated selective autophagic degradation

**DOI:** 10.1371/journal.ppat.1009300

**Published:** 2021-02-12

**Authors:** Yan Zeng, Shuai Xu, Yanli Wei, Xuegang Zhang, Qian Wang, Yane Jia, Wanbing Wang, Lu Han, Zhaoshan Chen, Zhengxiang Wang, Bo Zhang, Hualan Chen, Cao-Qi Lei, Qiyun Zhu

**Affiliations:** 1 State Key Laboratory of Veterinary Etiological Biology, Lanzhou Veterinary Research Institute, Chinese Academy of Agricultural Sciences, Lanzhou, China; 2 State Key Laboratory of Veterinary Biotechnology, Harbin Veterinary Research Institute, Chinese Academy of Agricultural Sciences, Harbin, China; 3 Hubei Key Laboratory of Cell Homeostasis, Frontier Science Center for Immunology and Metabolism, State Key Laboratory of Virology, College of Life Sciences, Wuhan University, Wuhan, China; Emory University School of Medicine, UNITED STATES

## Abstract

Influenza A virus (IAV) has evolved various strategies to counteract the innate immune response using different viral proteins. However, the mechanism is not fully elucidated. In this study, we identified the PB1 protein of H7N9 virus as a new negative regulator of virus- or poly(I:C)-stimulated IFN induction and specifically interacted with and destabilized MAVS. A subsequent study revealed that PB1 promoted E3 ligase RNF5 to catalyze K27-linked polyubiquitination of MAVS at Lys362 and Lys461. Moreover, we found that PB1 preferentially associated with a selective autophagic receptor neighbor of *BRCA1* (NBR1) that recognizes ubiquitinated MAVS and delivers it to autophagosomes for degradation. The degradation cascade mediated by PB1 facilitates H7N9 virus infection by blocking the RIG-I-MAVS-mediated innate signaling pathway. Taken together, these data uncover a negative regulatory mechanism involving the PB1-RNF5-MAVS-NBR1 axis and provide insights into an evasion strategy employed by influenza virus that involves selective autophagy and innate signaling pathways.

## Author summary

In 2013, H7N9 influenza viruses appeared in China and other countries resulting in 1, 567 human infections and 615 deaths. Understanding the cross-talk between virus and host is vital for the development of effective vaccines and therapeutics. Here, we identified the PB1 protein of H7N9 virus as a novel negative regulator that enhances the degradation of MAVS, an essential adaptor protein in the innate signaling pathway. Mechanistically, PB1 promoted the E3 ligase RNF5-mediated ubiquitination of MAVS and recruited the selective autophagic receptor NBR1 to associate with and deliver the ubiquitinated MAVS to the autophagosomes for degradation. Thus, the PB1-RNF5-MAVS-NBR1 axis inhibited innate immune antiviral response and facilitated virus replication by mediating MAVS degradation in an autophagosome-dependent manner. Our findings reveal a novel mechanism by which influenza virus negatively regulates the innate immune response.

## Introduction

Influenza A viruses (IAVs) are important zoonotic pathogens involved in global pandemics and annual epidemics, which are continually challenging animal and human health. IAVs (H1N1, H2N2, and H3N2) have caused four influenza pandemics since 1918 and an unprecedented number of deaths [1]. In the last two decades, highly pathogenic H5N1 and H7N9 IAVs emerged and continued to challenge animal and human health. H5Nx IAVs have infected more than 800 individuals across 16 countries, with an overall case fatality rate of 53% since 2003. Ten years later, the H7N9 viruses were first detected in live poultry markets in eastern China and spread to other countries and regions causing 1, 567 human infections and 615 deaths [2–5].

IAVs are segmented, single-stranded, negative-sense RNA viruses that are members of the *Orthomyxoviridae* family. The IAV genome consists of eight gene segments [basic polymerase 2 (PB2), basic polymerase 1 (PB1), acidic polymerase (PA), hemagglutinin (HA), nucleoprotein (NP), neuraminidase (NA), matrix (M), and nonstructural protein (NS)] that encode at least 14 proteins [6,7]. All eight viral RNA (vRNA) segments bind to the three RNA polymerases (PB2, PB1 and PA) and are encapsidated by NP to form viral ribonucleoprotein (vRNP) complexes[8]. The vRNP complex is the essential functional unit for the transcription and replication of the IAV genome [9,10]. PA, PB2, and PB1, including PB1-F2 which is a +1 frameshift product of PB1, participate in the regulation of innate immune responses and viral replication [11–17]. However, how the polymerase proteins of IAV modulate innate signaling is not fully understood.

The innate immune system is the first line of host defense against viral infection. During viral replication, viral nucleic acids present as pathogen-associated molecular patterns (PAMPs), which are detected by pattern recognition receptors (PRRs). Among the PRRs, the endosomal membrane-associated Toll-like receptor 3 (TLR3) recognizes extracellular and endosomal viral RNA in certain immune cells. However, in most cell types, cytosolic RNA is mainly detected by RIG-I-like receptors (RLRs), including retinoic acid-inducible gene-I (RIG-I) and melanoma differentiation-associated gene 5 (MDA5) [18]. The negative-sense RNA genome of IAV is strictly recognized by RLRs. Upon recognition of viral RNA through C-terminal RNA helicase domains, RIG-I undergoes conformational changes and interacts with the mitochondrial adaptor protein MAVS (also known as VISA, IPS-1, and Cardif) through its N-terminal CARD domain [19–22]. MAVS acts as a central platform for TRAF3 and TRAF6 engagement, which is followed by the activation of TBK1/IKKɛ and TAK1-IKK kinases and subsequent phosphorylation of the transcription factors IRF3 and NF-κB, and the ultimate induction of type I interferons (IFNs) and downstream antiviral genes [22]. Numerous studies have demonstrated that post-translational modifications (PTMs) are essential for the appropriate control of the innate immune response to RNA viruses [23–26]. The best characterized PTMs, ubiquitination and phosphorylation, have been reported to regulate innate antiviral immune responses via reversible PTMs of RIG-I, MAVS, and their downstream components [26]. However, to effectively infect and replicate in host cells, IAVs have evolved multiple strategies to counteract and evade host defenses. For instance, IAV NS1 inhibits IFN induction by blocking the activation of the retinoic acid-inducible gene I (RIG-I)-mediated signaling pathway and suppressing host gene expression [27–29]. PB2 impedes K63-linked polyubiquitination of TRAF3 and disrupts the formation of the MAVS–TRAF3 complex, thereby suppressing RIG-I signaling [30]. PB1-F2 affects MAVS-dependent signaling by employing various mechanisms [31–35]. However, whether other IAV proteins regulate the RIG-I-MAVS-mediated signal pathway has not been fully explored.

In eukaryotic cells, macroautophagy/autophagy is a conserved homeostatic process by which cells sequester damaged organelles, dysfunctional protein, and invading pathogens and then deliver them to lysosomes for degradation [36]. Recent studies indicate that autophagy can be highly selective and targets specific substrates through various cargo receptors that recognize specific degradation signals and selectively package the targets into autophagosomes for degradation [37–39]. In mammals, the most selective autophagy targeting signal is the modification of cargo with ubiquitin. Cargo receptors, such as polyubiquitin-binding protein sequestosome-1 (p62/SQSTM1), optineurin (OPTN), neighbor of *BRCA1* (NBR1), and nuclear dot protein 52 kD (NDP52), have both ubiquitin-binding domains and LC3-interacting regions (LIRs) [40]. Several selective autophagy receptors are reported to be involved in the modulation of immune responses; for example, the Nbr1 gene modulates p38 MAPK signaling in osteoblasts and is a regulator of osteoblast differentiation [41]. However, the cross-talk between autophagy and immune regulation during virus infection in mammals remains to be investigated.

In this study, we chose A/Environment/Suzhou/SZ19/2014 (SZ19), an early H7N9 strain, as a virus model and identified the PB1 protein as a novel negative regulator of RIG-I-mediated signaling. PB1 promoted E3 ligase RNF5-mediated ubiquitination of MAVS, which was recognized by the selective autophagy receptor NBR1 and further delivered to autophagosomes for degradation. Our findings reveal an important mechanism of influenza virus evasion of the host antiviral immune response.

## Results

### H7N9 PB1 inhibits RIG-I-mediated signaling

**Fig 1:**
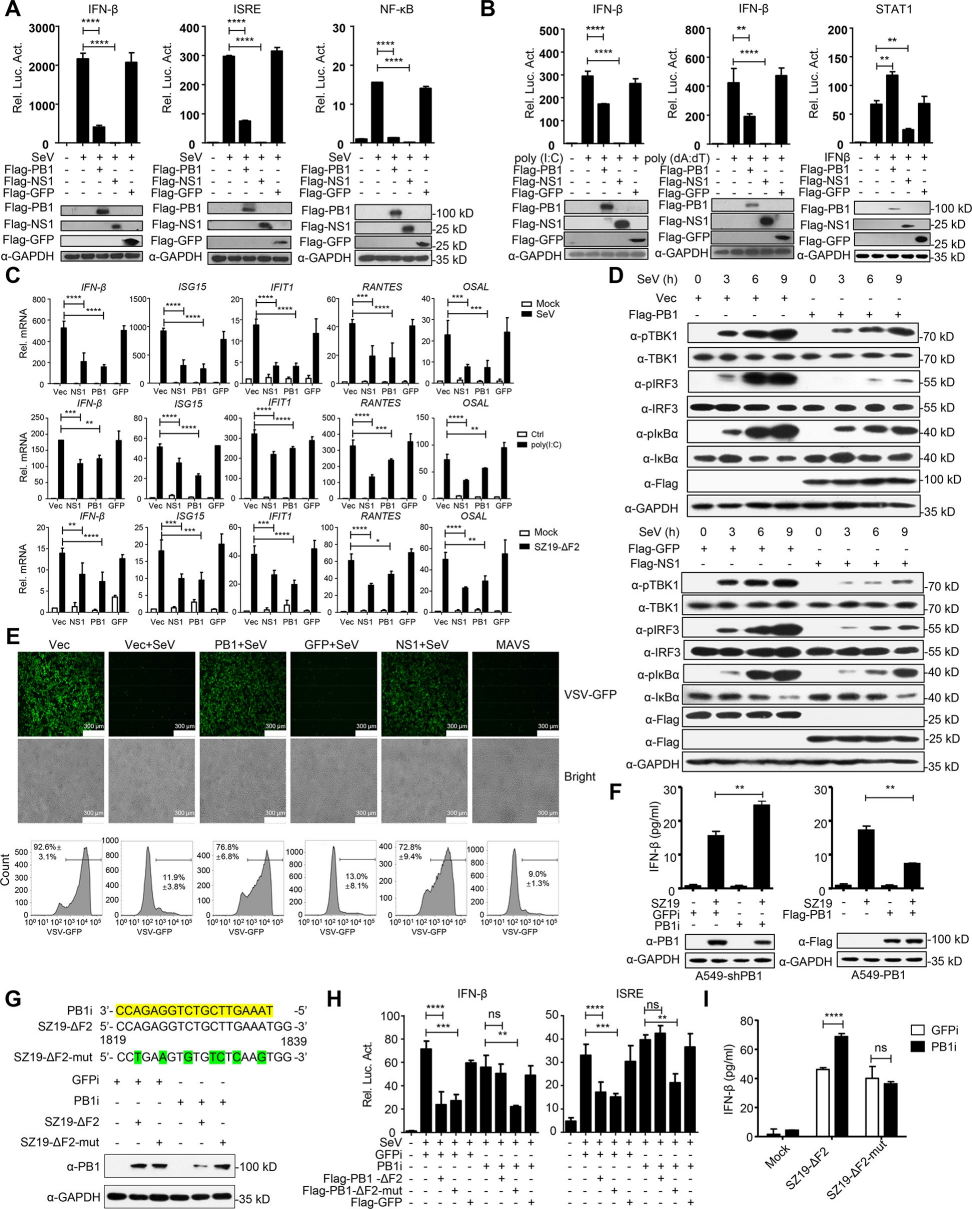
H7N9 PB1 negatively regulates type I IFN signaling pathway. (A) PB1 inhibits SeV-triggered activation of IFN-β promoter, ISRE and NF-κB. HEK293 cells were transfected with IFN-β promoter, ISRE and NF-κB luciferase reporter and the indicated plasmids (Flag-NS1 and Flag-GFP were chosen as positive and negative controls, respectively, throughout the study). Twenty hours after transfection, cells were left uninfected or infected with SeV for 12 h before reporter assays. (B) PB1 specifically inhibits cytosolic poly(I:C)- and poly(dA-dT)-triggered activation of IFN-β promoter. HEK293 cells were transfected with the IFN-β promoter or STAT1 luciferase reporter and the indicated plasmids. Twenty hours after transfection, the cells were left untreated or treated with poly(I:C), poly(dA:dT) and IFN-β for 12 h before use in reporter assays. (C) PB1 inhibits SeV-, poly(I:C)- and SZ19-ΔF2-triggered transcription of *IFN-β* and downstream genes. A549 cells transfected with the indicated plasmids were infected with SeV (MOI = 1) or SZ19-ΔF2 (MOI = 5) or were transfected with poly(I:C) for 12 h before qPCR analysis. (D) PB1 inhibits virus-induced phosphorylation of TBK1, IRF3, and IκBα. A459 cells were transfected with PB1, NS1, GFP plasmid, or a vector control. Twenty hours after transfection, the cells were infected with SeV (MOI = 1) for the indicated times. Cell lysates were separated by SDS-PAGE and analyzed by immunoblotting with the indicated antibodies. (E) PB1 facilitates VSV-GFP replication. HEK293 cells were transfected with indicated plasmids for 24 h, the cells then were infected with SeV (MOI = 1) for 24 h. The supernatants were inactivated by ultraviolet radiation and were collected to treat fresh HEK293 cells for another 24 h. The cells then were infected with VSV-GFP (MOI = 0.01) for 12 h, the cells were observed microscopically and then assessed by flow cytometry. Data are representative of three independent experiments [mean ± SD]. (F) The effects of knockdown or overexpression of PB1 on SZ19-induced IFN-β secretion. PB1-knocked down or overexpressed A549 cells were infected with SZ19 virus (MOI = 5) for 12 h before analysis of IFN-β secretion by ELISA. The lower immunoblots showed the expression levels of PB1 protein with the GAPDH control. (G) The generation of a mutant influenza virus SZ19-ΔF2-mut. PB1 was replaced with an RNAi off-target PB1 mutant with a 7-nucleotide nonsense mutation in the target sequence of the PB1-RNAi plasmids. The PB1-RNAi sequence is highlighted in yellow. The mutated nucleotides are shown in green. PB1-knockdown cells were infected with SZ19-ΔF2 or SZ19-ΔF2-mut virus for 24 h before immunoblotting analysis. (H) HEK293 cells were transfected with GFP-RNAi and PB1-RNAi along with the indicated plasmids. Twenty hours after transfection, the cells were left uninfected or infected with SeV for 12 h before use in reporter assays. (I) PB1-knockdown A549 cells were infected with SZ19-ΔF2 or SZ19-ΔF2-mut virus (MOI = 3) for 12 h before analysis of IFN-β secretion by ELISA. The data shown represent three independent experiments; bars represent the mean ± SD of the three independent experiments (n = 3). [*P*< 0.05(*), *P* < 0.01(**), *P* < 0.001(***), *P* < 0.0001(****); ‘ns’ indicates no significant difference].

To elucidate H7N9 viral proteins that may regulate RIG-I-mediated induction of downstream antiviral genes, we constructed 12 Flag-tagged expression plasmids encoding the individual H7N9 IAV proteins (PB2, PB1, PA, HA, NP, NA, M1, M2, NS1, NS2, PB1-F2, and PA-X). The PB1 gene was mutated to stop PB1-F2 expression by use of site-directed mutagenesis as described previously [42] and the mutant virus (SZ19-ΔF2) was rescued by using reverse genetics (S1A Fig). We then screened their ability to regulate the activation of the IFN-β promoter triggered by Sendai virus (SeV) infection or transfected cytoplasmic poly(I:C), a synthetic analog of viral dsRNA. This screen showed that most H7N9 viral proteins, but not M2 and NP, inhibited SeV-, or poly(I:C)-induced activation of the IFN-β promoter (S1B Fig). In the present study, we sought to explore how PB1 negatively regulates virus-triggered IFN-β activation. As shown in Fig 1A, overexpression of PB1 strongly inhibited SeV-triggered activation of the IFN-β promoter, ISRE, and NF-κB. Overexpression of PB1 also inhibited transfected cytoplasmic poly(I:C)- and poly(dA:dT)-triggered activation of IFN-β promoter, but had no inhibitory effects on STAT1 activation triggered by IFN-β (Fig 1B). Quantitative PCR (qPCR) analysis indicated that overexpression of PB1 significantly inhibited SeV-, cytoplasmic poly(I:C), and SZ19-ΔF2 -triggered transcription of the *IFNB1*, *ISG15* (IFN-stimulated gene 15), *IFIT1* (Interferon-induced protein with tetratricopeptide repeats 1), *RANTES* (Regulated upon activation normal T cell expressed and secreted factor), and *OASL* (Oligoadenylate synthetase-like protein) genes in human A549 and HEK293 cells (Figs 1C and S1C). In addition, we found that the PB1 protein of H1N1, H5N1, and H9N2 influenza viruses also markedly inhibited SeV-triggered induction of the *IFNB1*, *ISG15*, *IFIT1*, and *RANTES* genes in A549 cells, suggesting a general inhibitory effect of PB1 genes from different influenza virus subtypes (S1D Fig). SeV-induced phosphorylation of TBK1, IRF3, and IκBα, which are hallmarks of IRF3 and NF-κB activation, were markedly inhibited by PB1 (Fig 1D). Since PB1 inhibited virus-triggered induction of downstream effector genes, we next investigated whether PB1 plays a role in the cellular antiviral response. As shown in Fig 1E, ultraviolet radiation-inactivated supernatants from PB1-transfected HEK293 cells which were infected with SeV could not completely inhibit the replication of GFP-expressing vesicular stomatitis virus (VSV-GFP) as monitored by the GFP signal, which suggested that PB1 dampened the secretion of antiviral factors induced by SeV. To confirm the role of endogenous PB1 in the innate immune response, we generated a PB1-RNAi stable A549 cell line. An ELISA analysis indicated that knockdown of PB1 potentiated SZ19-triggered production of IFN-β in A549 cells. In contrast, overexpression of PB1 inhibited SZ19-triggered production of IFN-β in A549 cells (Fig 1F). To verify the specificity of the effect of PB1 on the type I IFN signaling pathway and to rule out the production of anomalous RNAs as stimuli, we generated a PB1-F2-mutant with a 7-nucleotide nonsense mutation in the target sequence of the PB1-RNAi plasmids (PB1-ΔF2-mut) and a second mutant virus (SZ19-ΔF2-mut) by use of reverse genetics (Fig 1G). Reporter assays indicated that PB1-ΔF2-mut, but not PB1-ΔF2, dramatically inhibited SeV-triggered activation of the IFN-β promoter and ISRE in PB1-RNAi stably transfected cells (Fig 1H). Consistent with this finding, knockdown of PB1 significantly potentiated the induction of IFN-β triggered by SZ19-ΔF2, but not SZ19-ΔF2-mut infection (Fig 1I). Taken together, these data suggest that PB1 protein is a generally negative regulator of the IAV-triggered antiviral response.

### H7N9 PB1 specifically interacts with MAVS

**Fig 2:**
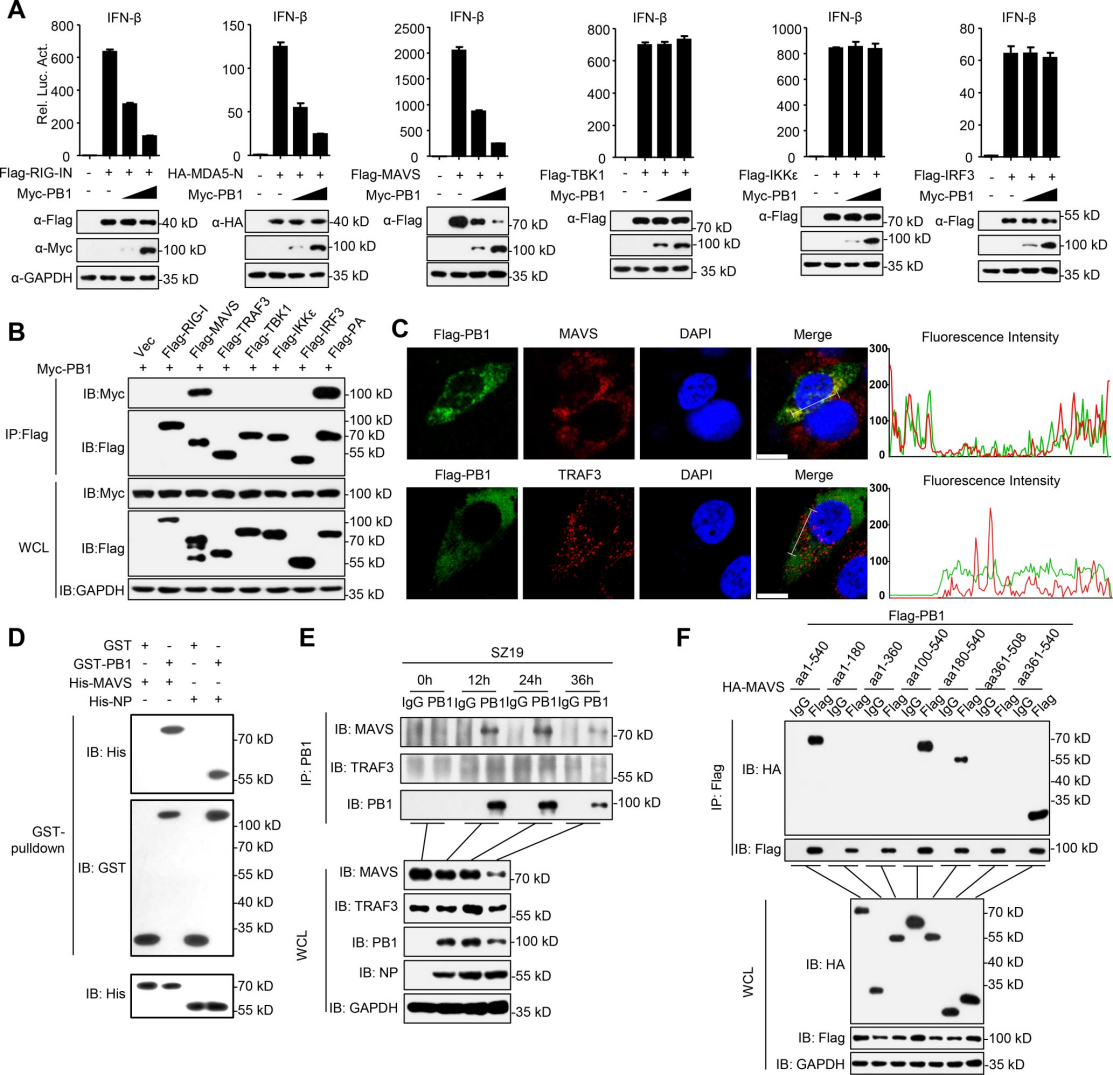
H7N9 PB1 specifically interacts MAVS. (A) PB1 inhibits activation of IFN-β promoter induced by RIG-I-CARD (indicated as RIG-IN), MDA5-CARD (indicated as MDA5-N) and MAVS. HEK293 cells were transfected with the indicated plasmids along with control vector or increased amounts of PB1 expression plasmids. Reporter assays were performed 24 h after transfection. (B) Overexpressed PB1 interacts with MAVS. HEK293 cells were transfected with the indicated plasmids for 24 h. Then the co-immunoprecipitation and immunoblotting analyses were performed with the indicated antibodies. (C) PB1 co-localizes with MAVS in the cell cytoplasm. A549 cells were transfected with a Flag-PB1 plasmid for 24 h, then stained with Flag antibody or MAVS/TRAF3 primary antibody and secondary antibody. The nuclei were stained by DAPI. The fluorescence intensity profile of DAPI (blue), Flag-PB1 (green) and MAVS/TRAF3 (red) was measured along the line drawn by Image J. Scale bars, 10 μm. (D) PB1 interacts with MAVS directly. Purified GST-PB1 was used to pull down purified His-MAVS. His-NP served as a control. (E) Endogenous PB1 is associated with MAVS. A549 cells were infected with SZ19 virus (MOI = 0.1) for the indicated times before coimmunoprecipitation and immunoblot analysis. (F) MAVS associates with PB1 through its transmembrane domain. HEK293 cells were transfected with the indicated plasmids before co-immunoprecipitation and immunoblotting analysis with the indicated antibodies. The data shown represent three independent experiments; bars represent the mean ± SD of the three independent experiments (n = 3).

We next investigated how PB1 inhibits the virus-induced antiviral response. To determine the molecular order of PB1 in the regulation of virus-triggered IFN-β activation, we transfected plasmids encoding RIG-I-CARD (RIG-IN), MDA5-CARD (MDA5-N), MAVS, TBK1, IKKε, or IRF3 together with the IFN-β promoter in the presence or absence of PB1. We found that overexpression of PB1 inhibited the activation of the IFN-β promoter triggered by the expression of RIG-IN, MDA5-N, and MAVS (but not TBK1, IKKε, and IRF3) in a dose-dependent manner (Fig 2A). Co-immunoprecipitation (co-IP) experiments indicated that Flag-tagged MAVS, but not other tested molecules specifically interacted with Myc-tagged PB1 (Fig 2B). Confocal microscopy confirmed that MAVS and PB1 co-localized in the cytoplasm (Fig 2C). Pull-down assays showed that GST-PB1 directly interacted with His-MAVS in vitro (Fig 2D). Endogenous co-IP experiments further verified that PB1 associated with MAVS following SZ19 virus infection at the indicated times (Fig 2E). Domain mapping experiments indicated that MAVS interacted with PB1 through its transmembrane domain (Figs 2F and S2A). Collectively, these data demonstrate that MAVS is the specific target of the PB1 protein of influenza viruses.

### PB1 mediates autophagic degradation of MAVS

**Fig 3:**
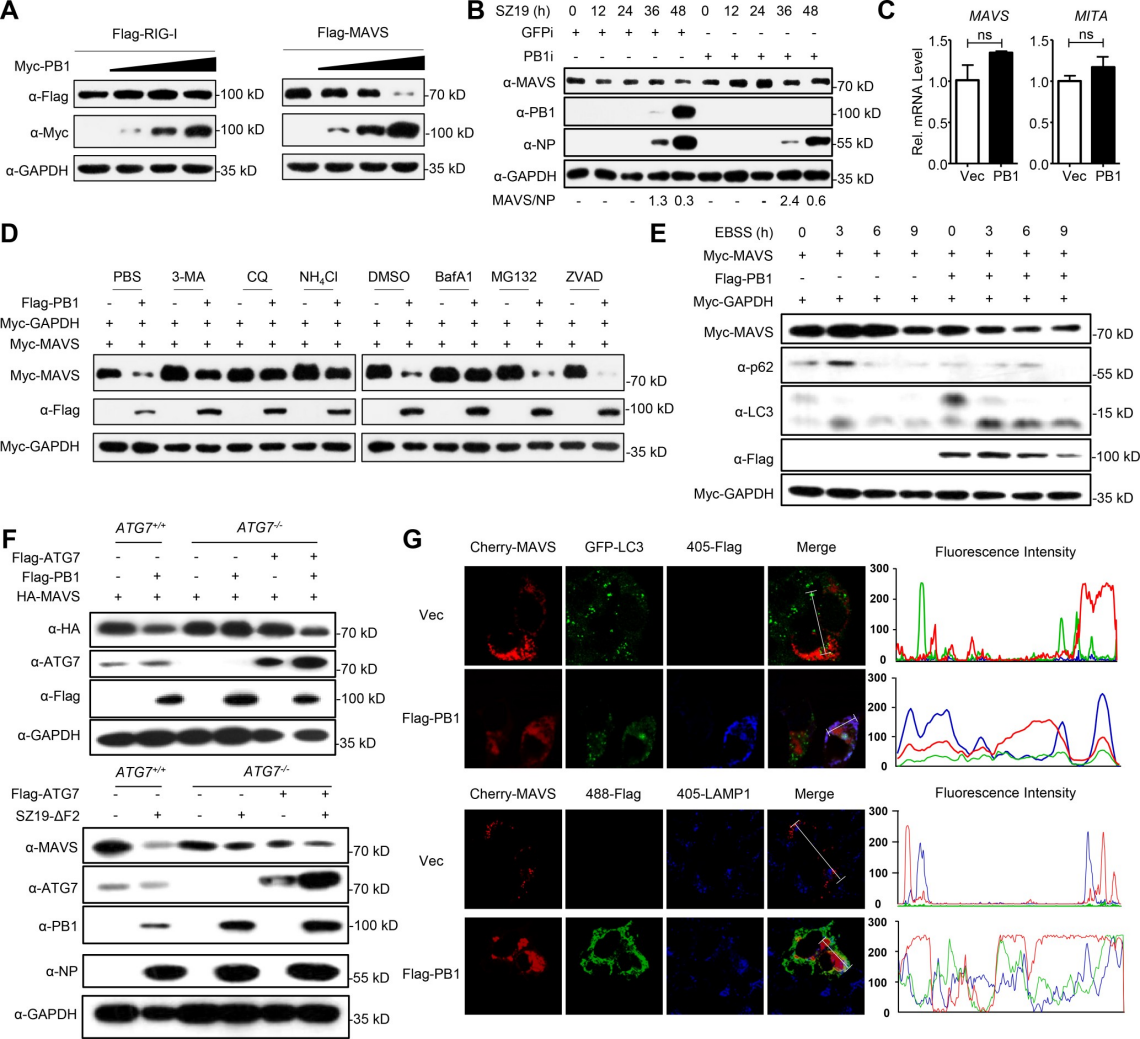
H7N9 PB1 promotes autophagic degradation of MAVS. (A) Overexpression of PB1 decreases the MAVS protein level. HEK293 cells transfected with Flag-MAVS or Flag-RIG-I with the increased amount of Myc-PB1 for 24 h before immunoblotting analysis. (B) PB1 decreases endogenous MAVS expression. A549 cells stably transduced with GFP-RNAi or PB1-RNAi were left uninfected or infected with SZ19 virus (MOI = 0.1) for the indicated times before immunoblot analysis with the indicated antibodies. The intensities of the indicated protein bands were determined by using image J, were normalized to NP, and are shown as the fold-change of MAVS/NP. (C) PB1 has no effect on MAVS mRNA expression. HEK293 cells were transfected with vector control or the PB1 plasmid for 24 h before qPCR analysis. (D) 3-MA, CQ, NH_4_Cl, and BafA1 blocked PB1-mediated MAVS degradation. HEK293 cells were transfected with the indicated plasmids for 20 h and then treated with 3-methyladenine (3-MA) (10 mM), chloroquine (CQ) (50 μM), NH_4_Cl (20 mM), MG132 (10 μM), bafilomycin A1 (BafA1) (0.2 μM), or ZVAD (20 μM) for 6 h. The cell lysates were then analyzed by immunoblotting with the indicated antibodies. (E) Activated autophagy increases PB1-mediated MAVS degradation. HEK293 cells were transfected with a plasmid encoding Myc-MAVS and Myc-GAPDH with or without Flag-PB1. At 24 h post-transfection, cells were treated with EBSS at the indicated time points and followed by immunoblot analysis. (F) ATG7 deficiency inhibits Flag-PB1- or SZ19-ΔF2 virus-mediated degradation of MAVS. Wild-type HEK293 cells or ATG7-deficient cells were transfected with the indicated plasmids for 24 h (up), or subsequently the cells were infected with SZ19-ΔF2 virus for another 24 h (down), followed by immunoblot analysis with the indicated antibodies. (G) PB1 co-localizes with MAVS at the autophagosomes and lysosomes. HEK293 cells were transfected with an empty vector or Flag-PB1 and the indicated plasmids. Twenty hours later, the cells were fixed with 4% paraformaldehyde and stained with anti-LAMP1 and / or anti-Flag before confocal microscopy. The fluorescence intensity profile of Cherry-MAVS (red), GFP-LC3 (green), and Flag (blue) or Cherry-MAVS (red), Flag (green), and LAMP1 (blue) was measured along the line drawn by Image J. The data shown represent three independent experiments; bars represent the mean ± SD of the three independent experiments (n = 3). (‘ns’ indicates no significant difference).

We repeatedly observed that overexpression of PB1 down-regulated the expression of MAVS (Fig 2A). We therefore hypothesized that PB1 might regulate the stability of MAVS. To test this hypothesis, we co-transfected Myc-tagged PB1 together with Flag-tagged MAVS or RIG-I and performed immunoblot analysis. PB1 specifically down-regulated the expression of MAVS, but not of RIG-I (Fig 3A). In addition, the PB1 protein from H1N1, H5N1, and H9N2 influenza viruses down-regulated the expression of MAVS (S2B Fig). We also found that knockdown of PB1 slowed the degradation of endogenous MAVS after SZ19 virus infection (Fig 3B). In contrast, PB1 did not affect the transcription of MAVS (Fig 3C). These data suggest that PB1 specifically destabilizes the MAVS protein.

The ubiquitin-proteasome and autophagy-lysosome pathways are major systems that control protein degradation in eukaryotic cells [43]. We found that PB1-mediated MAVS degradation could be mostly restored by treatment with the autophagy inhibitors 3-methylademine (3-MA), chloroquine (CQ), or bafilomycin A1 (Baf A1) and the lysosome inhibitor ammonium chloride (NH_4_Cl), but not the proteasome inhibitor MG132 or the caspase inhibitor ZVAD (Fig 3D). Moreover, PB1-mediated MAVS degradation was enhanced by starvation-induced autophagy activation under Earle’s balanced salt solution (EBSS) culture conditions (Fig 3E). Autophagy-related gene 7 (ATG7) is a key adaptor for autophagy degradation [44]. We found that ATG7 deficiency completely blocked PB1-mediated degradation of MAVS, which was fully restored by reconstitution with ATG7 (Fig 3F). In addition, ATG7 deficiency attenuated virus-induced degradation of MAVS, and reconstitution of ATG7 in *ATG7*^*-/-*^ cells restored the degradation of MAVS following SZ19-ΔF2 infection (Fig 3F). Confocal microscopy indicated that PB1 co-localized with MAVS at autophagosomes and lysosomes (Fig 3G). Green fluorescent protein (GFP) is sensitive to acidic or proteolytic conditions and can diminish quickly in acidic pH, whereas red fluorescent protein (RFP) is more pH stable [45]. The results of tandem mRFP-GFP-LC3 transfection experiments indicated that EBSS treatment and expression of PB1 induced autolysosome formation in HeLa cells (S2C Fig). Collectively, these results suggest that PB1 mediates the degradation of MAVS in an autophagy-dependent manner.

### PB1 promotes NBR1-mediated selective autophagic degradation of MAVS

**Fig 4:**
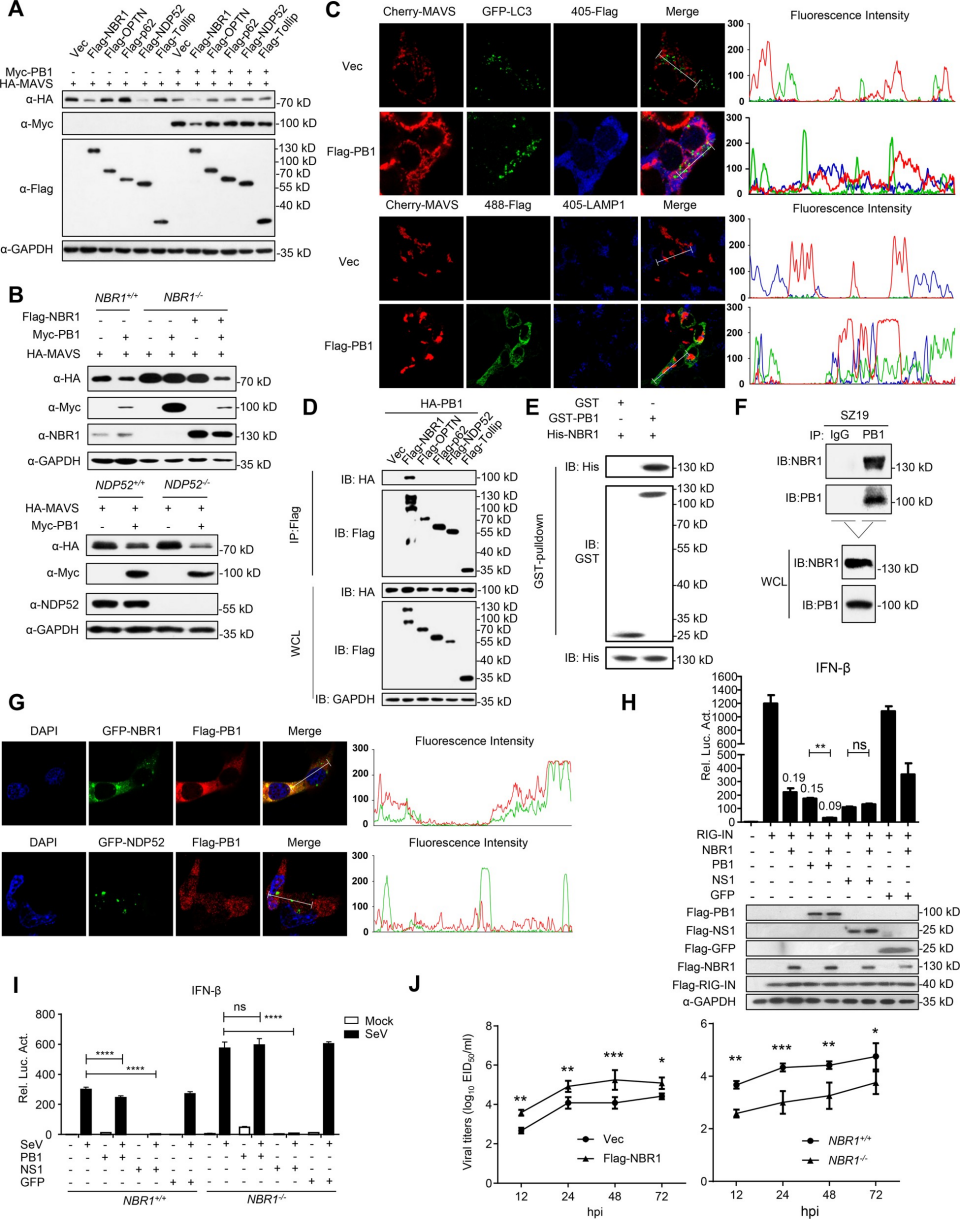
H7N9 PB1 potentiates the cargo receptor NBR1-mediated selective autophagic degradation of MAVS. (A) PB1 promotes NBR1-mediated degradation of MAVS. HEK293 cells were transfected with HA-MAVS and Flag-tagged cargo receptors (NBR1, OPTN, p62, NDP52, and Tollip) in the presence or absence of Myc-PB1for 24 h before immunoblotting analysis. (B) NBR1-deficiency inhibits PB1-mediated degradation of MAVS. NBR1- or NDP52-deficient cells were transfected with indicated plasmids for 24 h before immunoblotting analysis. (C) NBR1-deficiency impairs co-localization of PB1with MAVS at the autophagosomes and lysosomes. *NBR1*^*-/-*^ HEK293 cells were transfected with an empty vector or Flag-PB1 and the indicated plasmids. Twenty hours later, the cells were fixed with 4% paraformaldehyde and stained with anti-LAMP1 and / or anti-Flag before confocal microscopy. The fluorescence intensity profile of Cherry-MAVS (red), GFP-LC3 (green), and Flag (blue) or Cherry-MAVS (red), Flag (green) and LAMP1 (blue) was measured along the line drawn by Image J. (D) PB1 interacts with NBR1. HEK293 cells were transfected with the indicated plasmids for 24 h. Then the co-immunoprecipitation and immunoblotting analysis were performed. (E) PB1 interacts with NBR1 directly. Purified GST-PB1 was used to pull down purified His-NBR1. The immunoprecipitants were analyzed by immunoblotting. (F) Endogenous PB1 interacts with NBR1 following SZ19 infection. A549 cells were left uninfected or were infected with SZ19 virus (MOI = 0.1) for 24 h before coimmunoprecipitation and immunoblot analyses. (G) PB1 localizes with NBR1 in the cell cytoplasm. HeLa cells were transfected with GFP-NBR1/NDP52 and Flag-PB1 plasmids followed by DAPI staining and confocal microscopic observation. The fluorescence intensity profile of DAPI (blue), GFP-NBR1/NDP52 (green), and Flag-PB1 (red) was measured along the line drawn by Image J. (H) PB1 and NBR1 collaboratively inhibit the activation of IFN-β promoter induced by expression of RIG-I-CARD. HEK293 cells were transfected with the IFN-β promoter luciferase reporter and the indicated plasmids. Reporter assays were performed 24 h after transfection. The values on the different columns show the fold-change relative to the activity induced by RIG-IN. Luciferase assays were performed similarly as in 2A. (I) The effects of NBR1-deficiency on PB1-mediated inhibition of SeV-triggered activation of IFN-β promoter. *NBR1*^*-/-*^ and control cells were transfected with the indicated plasmids. Twenty hours after transfection, the cells were left uninfected or were infected with SeV for 12 h before being used in reporter assays. (J) The effects of overexpression or knockout of *NBR1* on SZ19-ΔF2 virus replication. HEK293 cells were transfected with control vector or PB1 plasmid for 24h. The cells were then infected with SZ19-ΔF2 virus (MOI = 0.01) for indicated time. The supernatants were harvested for virus titration (EID_50_/ml) (left). *NBR1*^*-/-*^ or control cells were infected with SZ19-ΔF2 virus (MOI = 0.01) for 36 h. The supernatants were harvested for virus titration (EID_50_/ml) (right). The data shown represent three independent experiments; bars represent the mean ± SD of the three independent experiments (n = 3). [*P*< 0.05(*), *P* < 0.01(**), *P* < 0.001(***), *P* < 0.0001(****); ‘ns’ indicates no significant difference].

The autophagic pathways which can be distinguished as the canonical or the selective autophagic pathway, play essential roles in degradation of intracellular proteins and organelles. It has been shown that ULK1/2, ATG13, FIP200, and ATG101 are essential for the initiation of the classical autophagic pathway [38,46]. We found that knockdown of ULK1, ATG13, FIP200, or ATG101 had no marked effect on the PB1-mediated degradation of MAVS (S3A Fig), suggesting that the PB1-mediated degradation of MAVS may not occur via the canonical autophagic pathway. In the non-canonical autophagic pathway, the cargo receptors deliver substrates to autophagosomes for the selective degradation. Having observed MAVS localized in autophagosomes and lysosomes (Fig 3G), we hypothesized that specific cargo receptors may deliver MAVS to the autophagosomes for degradation. Co-IP experiments indicated that MAVS interacted with NBR1, OPTN, p62, and NDP52, but not with Tollip (S3B Fig), and only overexpression of NBR1 and NDP52 downregulated the expression of MAVS (Fig 4A). Interestingly, overexpression of PB1 potentiated MAVS degradation mediated by NBR1 but not NDP52 (Fig 4A). In contrast, loss of NBR1, but not NDP52, restored PB1-mediated degradation of MAVS (Fig 4B). Furthermore, an immunofluorescence assay showed that PB1 failed to co-localize with MAVS in autophagosomes or lysosomes in NBR1-deficient cells (Fig 4C). In addition, co-IP experiments showed that PB1 specifically interacted with NBR1 but not with other cargo receptors (Fig 4D). Pull-down analysis indicated that GST-PB1 directly interacted with His-NBR1 (Fig 4E), and Endogenous co-IP experiments indicated that SZ19 PB1 physiologically associated with NBR1 following SZ19 virus infection (Fig 4F). In addition, confocal microscopy suggested that PB1 co-localized with NBR1 but not with NDP52 in the cytoplasm (Fig 4G). Consistent with these observations, luciferase assays indicated that overexpression of NBR1 significantly inhibited RIG-IN-mediated activation of the IFN-β promoter, which was further attenuated in the present of PB1 (Fig 4H). As shown in Fig 4I, PB1 could not inhibit SeV-triggered activation of the IFN-β promoter in NBR1-deficient cells compared with control cells. In the same experiments, the NS1 protein of IAV inhibited activation of the IFN-β promoter triggered by SeV in both control and NBR1-deficient cells. At the virus level, overexpression of NBR1 enhanced SZ19-ΔF2 virus replication, whereas NBR1-deficiency significantly inhibited viral replication compared to wild-type cells at the indicated times (Fig 4J). Collectively, these results suggest that PB1 promotes NBR1-mediated selective autophagic degradation of MAVS and facilitates SZ19 virus replication.

### PB1 increases the K27-linked ubiquitination of MAVS

**Fig 5:**
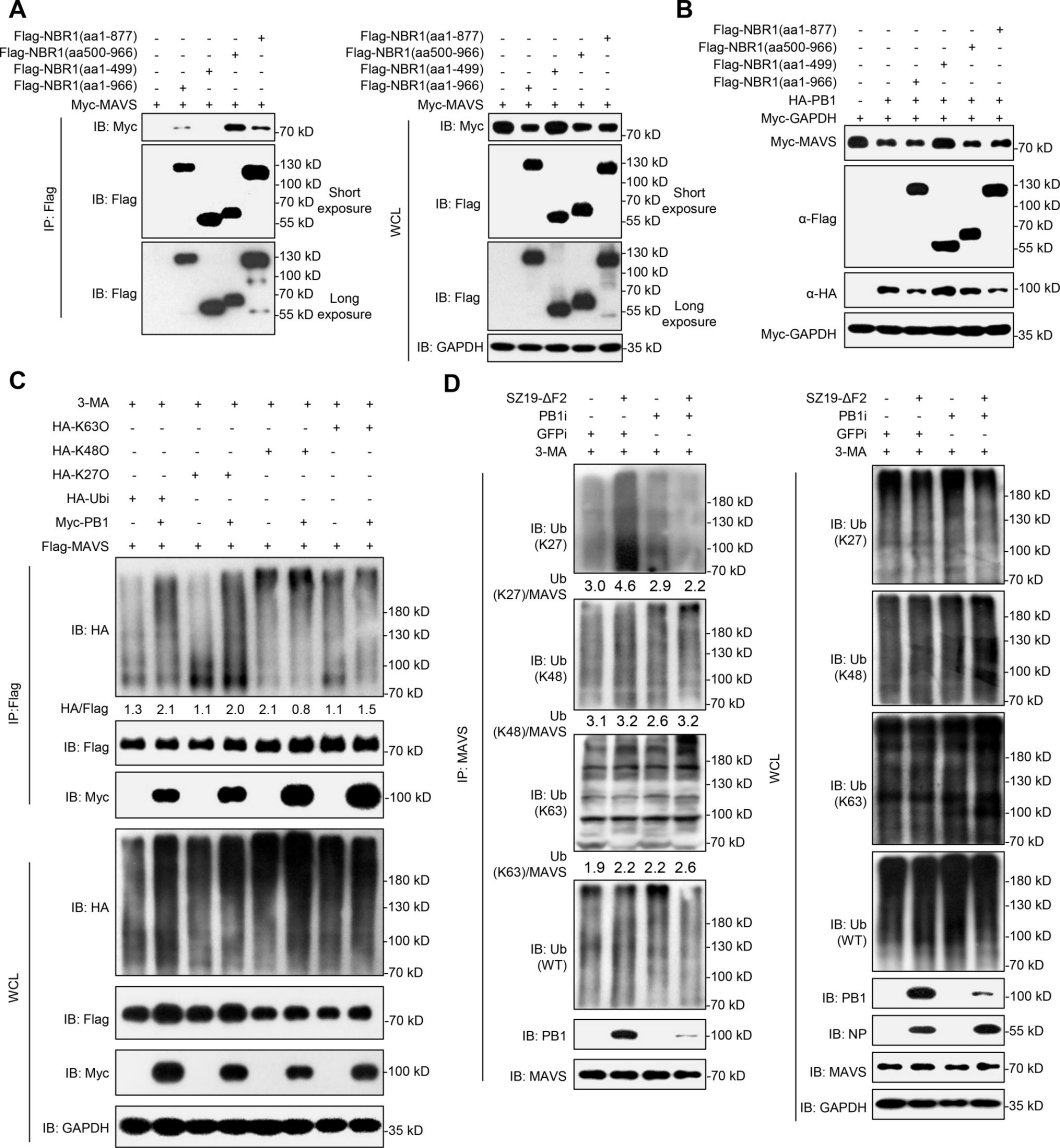
H7N9 PB1 promotes the K27-linked ubiquitination of MAVS. (A) MAVS interacts with NBR1. HEK293 cells were transfected with the indicated plasmids for 24 h. Then the co-immunoprecipitation and immunoblotting analysis were performed with the indicated antibodies. (B) NBR1 (aa500-966) is indispensable for MAVS degradation. HEK293 cells were transfected with HA-MAVS, Myc-PB1 and Flag-NBR1 with the indicated truncations for 24 h before immunoblot analysis with the indicated antibodies. (C) PB1 enhances K27-linked ubiquitination of MAVS. HEK293 cells transfected with Flag-MAVS, HA-ubiquitin, or its mutants [KO, which all but one lysine residues were simultaneously mutated to arginines (K-only)] together with a control and PB1 plasmids were pre-treated with 3-MA (10 mM) for 6h, the cells then were subjected to co-immunoprecipitation and immunoblotting analysis with the indicated antibodies. The intensities of the indicated protein bands were determined by using image J, were normalized to Flag-MAVS, and are shown as the fold-change of HA/Flag. (D) The effects of PB1-RNAi on SZ19-ΔF2 virus-triggered ubiquitination of endogenous MAVS. PB1-knockdown A549 cells were left uninfected or infected with SZ19-ΔF2 virus (MOI = 0.01) for 24 h. The cells then were treated with 3-MA for 6 h before ubiquitination assays with the indicated antibodies. The intensities of the indicated protein bands were determined by using image J, were normalized to MAVS, and are shown as the fold-change of the indicated Ub/MAVS. The data shown represent three independent experiments.

NBR1 exerts its biological effects mainly via two important functional domains: the LIR domain associates with LC3, and the UBA domain recognizes ubiquitinated substrates [47]. To identify which domain is required for MAVS degradation, we performed domain mapping experiments and found that the LIR domain and UBA domain of NBR1 associates with MAVS (Figs 5A and S3C). NBR1 lacking the LIR and UBA domains lost the ability to promote the PB1-mediated MAVS degradation (Fig 5B). Since ubiquitin chains attached to substrates serve as a major signal for recognition by cargo receptors, we next determined whether PB1 affects the ubiquitination of MAVS. We found that overexpression of PB1 potentiated MAVS ubiquitination. Specifically, overexpression of PB1 significantly increased K27-linked ubiquitination of MAVS, but did not potentiate K63- or K48-linked ubiquitination of MAVS (Fig 5C). Moreover, SZ19-ΔF2 virus infection specifically enhanced the K27-linked, but not K48- or K63-linked endogenous ubiquitination of MAVS, in contrast, knockdown of PB1 inhibited the SZ19-ΔF2-induced K27-linked, but not K48- or K63-linked endogenous ubiquitination of MAVS (Fig 5D). Taken together, these results suggest that PB1 promotes K27-linked ubiquitination and autophagic degradation of MAVS.

### PB1 promotes RNF5-mediated autophagic degradation of MAVS

**Fig 6:**
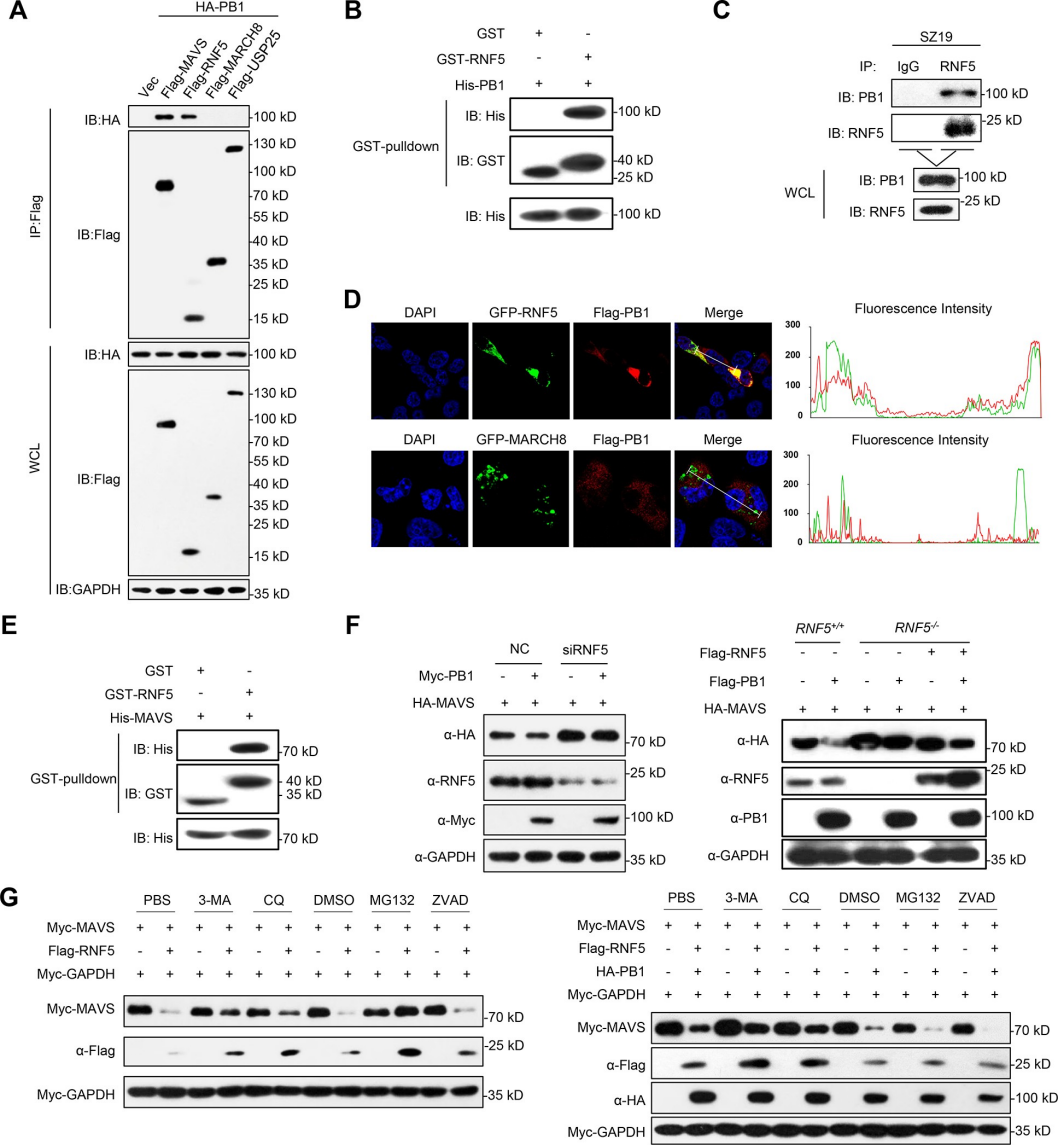
H7N9 PB1 facilitates RNF5 to degrade MAVS through the autophagosomal pathway. (A) Overexpression of PB1 interacts with RNF5. HEK293 cells were transfected with the indicated plasmids for 24 h before co-immunoprecipitation and immunoblotting analysis. (B) PB1 interacts with RNF5 directly. Purified GST-RNF5 was used to pull down purified His-PB1. The immunoprecipitants were analyzed by immunoblotting. (C) Endogenous PB1 interacts with RNF5 after SZ19 virus infection. A549 cells were left uninfected or were infected with SZ19 virus (MOI = 0.1) for 24 h before coimmunoprecipitation and immunoblot analysis. (D) PB1 co-localizes with RNF5 in the cytoplasm. HeLa cells transfected with Flag-PB1 along with the GFP-RNF5 or MARCH8 plasmids. Twenty hours later, the cells were fixed with 4% paraformaldehyde and stained with anti-Flag before confocal microscopy. The nuclei were stained by DAPI. The fluorescence intensity profile of DAPI (blue), GFP-RNF5/MARCH8 (green), and Flag-PB1 (red) was measured along the line drawn by Image J. (E) MAVS interacts with RNF5 directly. Purified GST-RNF5 was used to pull down purified His-MAVS. The immunoprecipitants were analyzed by immunoblotting. (F) RNF5-deficiency inhibits PB1-mediated degradation of MAVS. *RNF5* knockdown or knockout cells were transfected with the indicated plasmids for 24 h before immunoblotting analysis. (G) PB1 enhances RNF5-mediated autophagic degradation of MAVS. HEK293 cells were transfected with the indicated plasmids for 20 h. The cells then were treated with 3-MA (10 mM), CQ (50 μM), MG132 (10 μM), or ZVAD (20 μM) for another 6 h before immunoblot analysis. The data shown represent three independent experiments.

Because IAV PB1 has no E3 ubiquitin ligase activity, we hypothesized that PB1 might function as a scaffold to link MAVS to its E3 ligases for ubiquitination and degradation. Previous studies have identified E3 ubiquitin ligases that mediate MAVS degradation, including RNF5, and MARCH8 [39,48,49]. Co-IP experiments indicated that PB1 specifically interacted with RNF5 but not with MARCH8 or USP25, as a negative control (Fig 6A). Pull-down analysis indicated that His-PB1 directly interacted with GST-RNF5 (Fig 6B), and endogenous PB1 associated with RNF5 upon SZ19 virus infection (Fig 6C). In addition, confocal microscopy revealed that PB1 co-localized with RNF5 in the cytoplasm (Fig 6D). RNF5 is known to target MAVS for ubiquitination and degradation[48]. Pull-down assays confirmed that GST-RNF5 directly associates with His-MAVS in vitro (Fig 6E). We further found that knockdown of RNF5 inhibited PB1-mediated degradation of MAVS, RNF5 deficiency blocked the degradation of MAVS, but reconstitution of RNF5 into *RNF5*^*-/-*^ cells restored the degradation of MAVS (Fig 6F). RNF5-mediated degradation of MAVS was also inhibited by the proteasomal inhibitor MG132, as previously demonstrated elsewhere[48]. Unexpectedly, RNF5-mediated degradation of MAVS was also partially inhibited by the autophagic inhibitors 3-MA and CQ (Fig 6G). Interestingly, RNF5-mediated MAVS degradation was mainly blocked by 3-MA and CQ, and not MG132 in the presence of PB1 (Fig 6G). Together, these data show that PB1 promotes RNF5-mediated autophagic degradation of MAVS.

### PB1 potentiates RNF5-mediated K27-linked ubiquitination and degradation of MAVS

**Fig 7:**
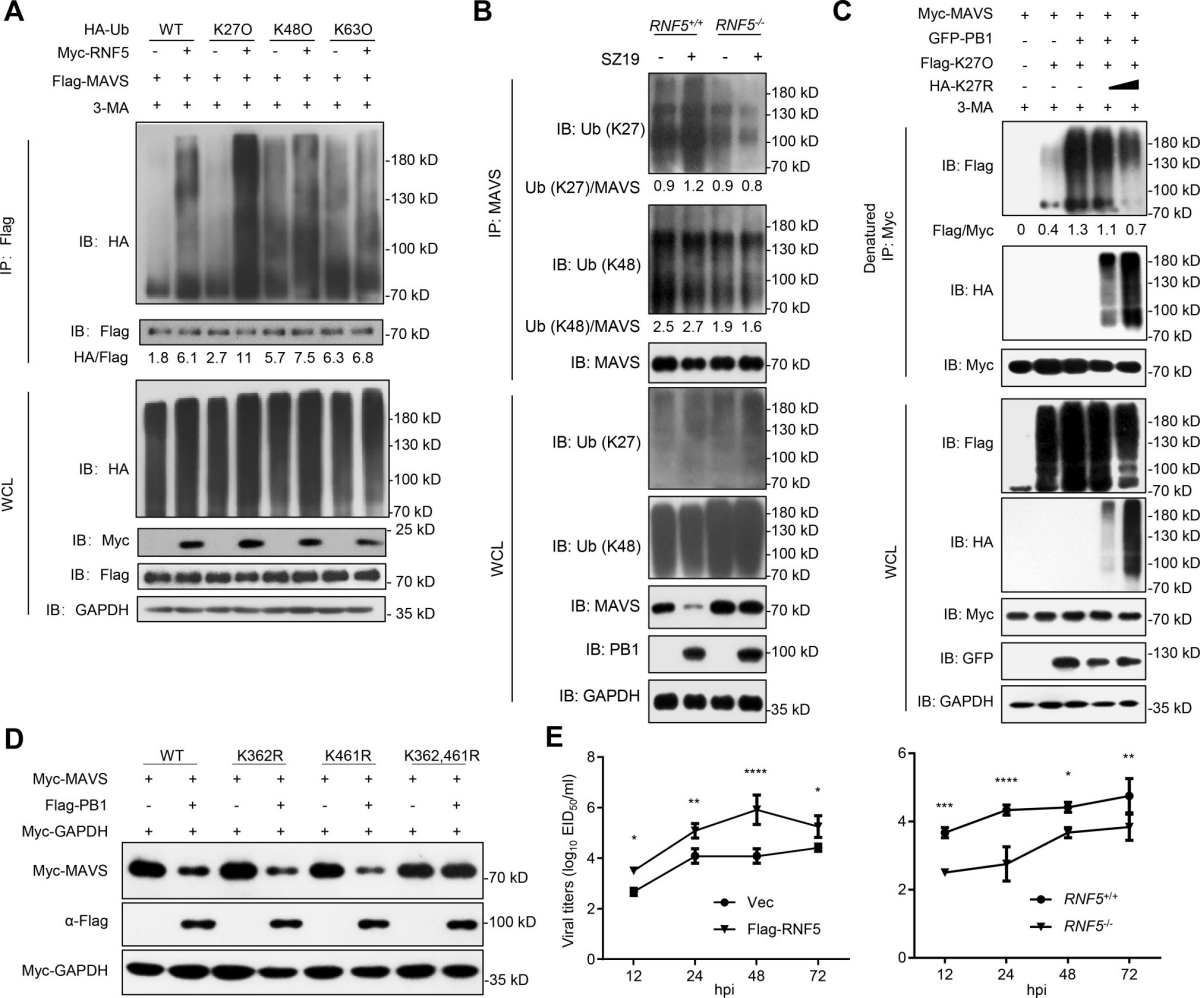
H7N9 PB1 facilitates RNF5 to ubiquitinate MAVS. (A) RNF5 catalyzes K27-linked ubiquitination of MAVS. HEK293 cells were transfected with Flag-MAVS, HA-ubiquitin, or its mutants (KO, K-only) together with a control and Myc-RNF5 plasmids for 20 h. The cells were then treated with 3-MA for 6 h before use in ubiquitination assays with the indicated antibodies. The intensities of the indicated protein bands were determined by using image J, were normalized to Flag-MAVS, and are shown as the fold-change of HA/Flag. (B) RNF5 deficiency inhibits SZ19-triggered K27-linked ubiquitination of MAVS. *RNF5*^*-/-*^ and control cells were infected with SZ19 virus (MOI = 0.1) for 24 h. The cells were then treated with 3-MA for 6 h before use in ubiquitination assays with the indicated antibodies. The intensities of the indicated protein bands were determined by using image J, were normalized to MAVS, and are shown as the fold-change of the indicated Ub/MAVS. (C) K27R ubiquitin inhibits PB1-mediated K27-linked ubiquitination of MAVS. HEK293 cells were transfected with Myc-MAVS, Flag-K27O, and HA-K27R (which K27 lysine residues was mutated to arginine) together with a control and GFP-PB1 plasmids for 20 h. The cells were then treated with 3-MA for 6 h before use in ubiquitination assays with the indicated antibodies. The intensities of the indicated protein bands were determined by using image J, were normalized to Myc-MAVS, and are shown as the fold-change of Flag/Myc. (D) MAVS bearing the K362 and 461R mutations is resistant to PB1-mediated degradation. HEK293 cells were transfected with Myc-MAVS and the indicated mutants in the presence or absence of Flag-PB1 for 24 h before immunoblot analysis. (E) The effects of overexpression or knockout of *RNF5* on SZ19-ΔF2 virus replication. HEK293 cells were transfected with control vector or RNF5 plasmid for 24 h. The cells were then infected with SZ19-ΔF2 virus (MOI = 0.01) for the indicated times. The supernatants were harvested for virus titration (EID_50_/ml) (left). *RNF5*^*-/-*^ or control cells were infected with SZ19-ΔF2 virus (MOI = 0.01) for the indicated times. The supernatants were harvested for virus titration (EID_50_/ml) (right). The data shown represent three independent experiments; bars represent the mean ± SD of the three independent experiments (n = 3). [*P*< 0.05(*), *P* < 0.01(**), *P* < 0.001(***), *P* < 0.0001(****)].

Next, we determined whether and how RNF5 mediated the K27-linked ubiquitination and degradation of MAVS. In a mammalian overexpression system, RNF5 strongly mediated K27-linked polyubiquitination of MAVS in the presence of 3-MA (Fig 7A). Conversely, SZ19-induced K27-linked polyubiquitination of MAVS was markedly decreased in RNF5-deficient cells compared with control cells (Fig 7B). We also examined the effects of K27R ubiquitin on PB1-induced ubiquitination of MAVS. As shown in Fig 7C, overexpression of K27R ubiquitin inhibited PB1-induced K27-linked ubiquitination of MAVS. We next investigated which lysine residues in MAVS are targeted by PB1. To this end, we mutated a series of lysines (K) of MAVS and examined their effects on PB1-mediated MAVS degradation as shown in Figs 7D and S4A. Only mutation of Lysine 362 and Lysine 461 to Arginine (K362/461R) abolished PB1-mediated degradation of MAVS (Fig 7D). Finally, we tested whether RNF5 influences H7N9 virus replication. Infection experiments at the indicated times showed that transient overexpression of RNF5 in HEK293 cells facilitated SZ19 viral replication, whereas viral titers were markedly reduced in RNF5-deficient cells (Fig 7E). Taken together, our results suggest that PB1 potentiates RNF5-mediated K27-linked polyubiquitination and autophagic degradation of MAVS at lysines 362 and 461.

## Discussion

MAVS plays vital roles in virus-triggered type I interferon induction and innate antiviral responses. Various studies have shown that post-translational modifications of MAVS are critical for its activation of the downstream signaling cascades [24,48,50,51]. Conversely, there is mounting evidence that certain viral proteins exert negative effects by targeting the MAVS molecule for immune evasion [52–56]. In the present study, we demonstrated that the PB1 protein of influenza A virus collaborates with the E3 ligase RNF5 via a stepwise biological process to mediate K27-linked polyubiquitination of MAVS. The ubiquitinated MAVS is then recognized by the selective autophagic receptor NBR1 in the presence of the PB1 protein, finally leading to the delivery of the PB1-RNF5-MAVS-NBR1 complex to the autophagosomes for degradation. In this way, the MAVS-mediated innate signaling pathway is disrupted and the interferon responses are suppressed, resulting in enhanced viral replication. The current study thus revealed a novel strategy mediated by the PB1 protein of influenza A virus in which the virus utilized the host autophagy system to degrade a critical molecule in the innate signaling pathway and blocked the type I interferon responses to promote virus survival.

Innate immune responses are the first line of defense against viral infection. However, to effectively enter and propagate in host cells, viruses have to combat and evade the host innate defenses by utilizing various strategies. Strategies used by different viruses include evasion of Toll-like receptor (TLR) / RIG-I–like receptor (RLR) recognition, cleavage or degradation of essential innate immune molecules, and blockade of molecular interactions [30,54,57–59]. In our study, we used SZ19 IAV (H7N9) isolated from the environment in Suzhou City, China in 2014 as a virus model and screened all the viral proteins of SZ19 by using the luciferase reporter assay. Among the 12 viral proteins, the molecular mechanism of PB1 mediated inhibition of the type I IFN signaling pathway is not yet fully understood, although several previous studies have reported that the PB1 of influenza A and B viruses has biological significance to the IFN signaling pathway [14,15]. Moreover, in the present study, we found that inhibition of IFN signaling mediated by PB1 is common to several different subtypes of influenza virus.

PB1 is an essential factor of the IAV RNA polymerase complex for viral transcription and replication. Live virus generated by reverse genetics is not viable in the absence of the PB1 gene. Therefore, we employed a knockdown strategy to decrease the expression of PB1 at the virus level for our functional study. Knockdown of PB1 could affect the viral polymerase activity and vRNA packing, leading to the attenuation of viral replication; however, in the present study, our data clearly indicate that PB1 inhibited type I interferon induction because the PB1 protein mediated the degradation of a critical component of the innate signaling pathway, specifically MAVS, but not RIG-I. Subsequently, we revealed that PB1 recruited NBR1, which is a selective autophagic receptor, to associate with the K27-linked ubiquitinated MAVS for degradation. Fu et al. previously reported that IAV PB1 proteins could be sensed and targeted by the host restrictor TRIM32 for ubiquitination and degradation, leading to the suppression of viral replication [60]. Our data suggested that PB1 mediates MAVS degradation for virus survival via a novel mechanism.

Ubiquitination is an important posttranslational modification for the dynamic modulation of signal transduction pathways [61]. RLR signal transduction is also regulated by ubiquitination upon RNA virus infection. Several studies have now demonstrated that MAVS could be modified by ubiquitination during viral infection [62–66]. The K48- and K63-linked ubiquitination of MAVS are well characterized. MAVS protein stability is regulated by several ubiquitin E3 ligases via K48-linked ubiquitination. RNF5, RNF125, AIP4/ITCH, Smurf1/2, and MARCH5 have been shown to mediate the ubiquitin-dependent degradation of MAVS, which may control excessive immune responses or be exploited by viruses to block the activation of innate signaling pathways. Paz et al. found that K63-linked ubiquitinated MAVS recruits the downstream IKKɛ to mitochondria following RNA virus infection, leading to NF-κB activation and IFNβ transcriptional activation [67]. Liu et al. reported that TRIM31 interacts with MAVS and promotes K63-linked polyubiquitination of MAVS at Lys10/Lys311/Lys461 after RNA virus infection, leading to enhancement of MAVS activation and downstream antiviral effects [66]. Ubiquitination is a dynamic process that is critical for fine-tuning the RLR-mediated antiviral response. In our study, we found the PB1 protein of SZ19 virus markedly enhanced the K27-linked ubiquitination of MAVS, but not K48-, or K63-linked ubiquitination of MAVS (Fig 5C and 5D). Subsequently, we identified RNF5 as an E3 ligase that interacts with MAVS and enhances its K48-linked ubiquitination, which is consistent with a previous study[48]. In addition, we observed that RNF5 could conjugate K27-linked ubiquitin moieties to MAVS (Fig 7A). It is interesting that RNF5-mediated K27-linked ubiquitination directly led to autophagic degradation of MAVS through NBR1 in this study (Fig 7C). Consistent with this finding, Jin et al. demonstrated that IFN-induced Tetherin recruits MARCH8 to mediate the K27-linked ubiquitination of MAVS for NDP52-dependent autophagic degradation [39]. In addition, Sparrer et al. reported that K27-linked ubiquitination in TRIM23 could activate TBK1-mediated selective autophagy [68]. Moreover, by mapping, we found that RNF5 mediated the K27-linked ubiquitination of MAVS at Lys362/Lys461 following SZ19 influenza virus infection (Figs 7D and S4A). Previously, Zhong et al. demonstrated that RNF5 targets MAVS and mediated a K48-linked ubiquitination of MAVS for degradation after viral infection[48]. While the K48- and K63-ubiquitin linkages have been well-studied, the K27-type linkage is so far considered an atypical ubiquitin linkage because its functions have not been investigated in much detail. In the present study, we revealed that RNF5 mediates MAVS ubiquitination at Lys362/Lys461 with K27-linked ubiquitination chains and that this process is enhanced by PB1 upon H7N9 influenza virus infection. Interestingly, the replication of SZ19 virus was substantially inhibited in *MAVS*^*-/-*^ cells reconstituted with wild-type but not degradation-resistant MAVS (MAVS K362/461R) (S4B Fig). It has been reported that TRIM31 catalyzes the K63-linked polyubiquitination of MAVS at K461, which promotes the formation of prion-like aggregates of MAVS and induces the innate antiviral response [66]. Although mutation of K362 and K461 of MAVS led to its degradation resistance, these mutations also abolished the formation of prion-like aggregates of MAVS, which is a prerequisite for MAVS-mediated signaling. This explains why reconstitution of the MAVS K362/461R mutant did not restore the antiviral activity of the *MAVS*^*-/-*^ cells (S4B Fig).

Regarding the influenza virus-related autophagic process, Gannage et al. first revealed that the influenza virus M2 protein blocks the autophagosome fusion with lysosomes to inhibit macroautophagy; however, this biological process does not affect the influenza virus replication [69]. Recently, Wang et al. found that the NP and M2 proteins of influenza A virus induce AKT-mTOR-dependent autophagy to benefit viral replication [70]. However, the same laboratory revealed that the M2 protein of influenza virus co-localized and interacted with MAVS on mitochondria, and positively regulated the MAVS-mediated innate immune response [71]. These studies primarily revealed the classical autophagy process during influenza virus infection. In the present study, we newly identified the PB1 protein from SZ19 influenza virus as being recruited and interacting with the selective autophagic receptor NBR1 to recognize the ubiquitinated MAVS for lysosomal degradation. Intriguingly, we found that the selective autophagic receptors NBR1 and NDP52 mediated MAVS degradation *in vitro*; however, only the NBR1 receptor was responsible for MAVS degradation in the presence of the PB1 protein (Fig 4A). This finding suggests that the PB1 protein preferentially interacts with NBR1, but not NDP52, to recognize the ubiquitinated MAVS. Our study is thus the first to demonstrate that the PB1 protein of influenza A virus induces MAVS degradation via the selective autophagic pathway.

**Fig 8:**
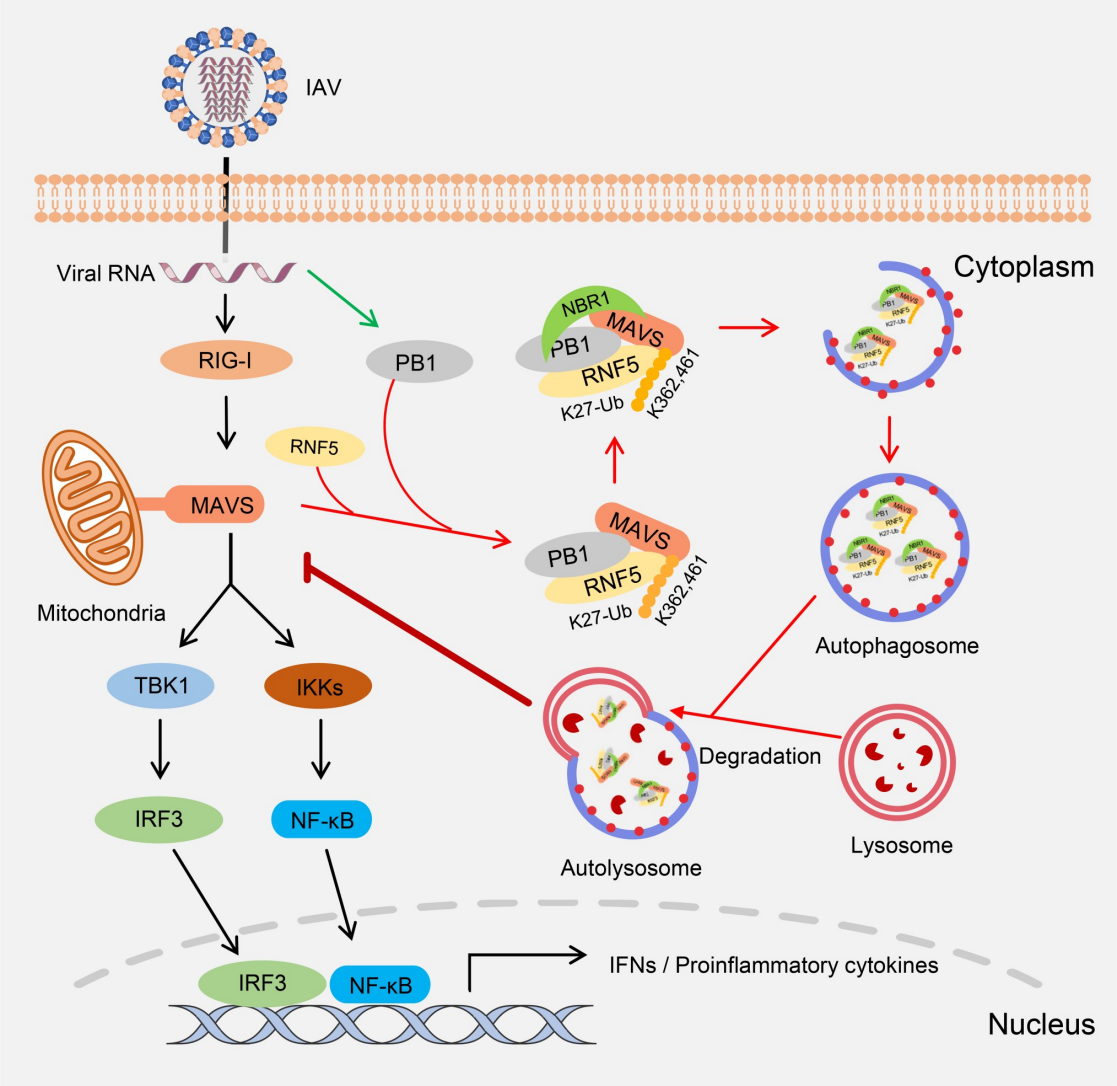
A working model of the role of the PB1 protein of influenza A virus in the regulation of MAVS-mediated signaling. Black arrows indicate RIG-I/MAVS mediated type I IFN signaling pathway; green arrow indicates the expression of PB1 protein; red arrows indicate the process of PB1-mediated autophagic degradation of MAVS.

Based on our data, we propose a working model for the regulatory role of the PB1 protein of H7N9 influenza A virus in the MAVS-mediated innate signaling pathway (Fig 8). Upon infection by influenza A virus, the viral genomic RNA is sensed by RIG-I, which activates the RLR-mediated signaling. To counteract the host antiviral immune response, the PB1 protein potentiates the K27-linked ubiquitination of MAVS mediated by the E3 ligase RNF5. In addition, the PB1 protein preferentially recruits and interacts with the selective autophagic receptor NBR1 to recognize the ubiquitinated MAVS. Subsequently, the PB1-RNF5-MAVS-NBR1 complex and autophagosomes fuse with the lysosomes for MAVS degradation. Finally, the MAVS-mediated innate signaling pathway is disrupted, leading to the suppression of type I interferon induction and facilitation of virus replication. Our findings suggest that the ubiquitination and autophagic degradation of MAVS mediated by the PB1 protein of influenza A virus is a novel regulatory mechanism by which influenza A virus evades the host antiviral immune response.

## Materials and methods

### Biosafety and ethical statements

All experiments with live influenza viruses were conducted within enhanced biosafety level 3+ (ABSL3+) facilities. The details of the facility and the biosafety and biosecurity measures used have been previously reported [72].

### Cells, viruses and plasmids

HEK293 cells (human embryonic kidney 293 cells, ATCC), were grown in Dulbecco’s modified Eagle’s medium (DMEM) supplemented with 10% fetal bovine serum (FBS) (Life Technologies), 100 U/ml penicillin, and 100 μg/ml streptomycin (Life Technologies). A549 cells (human lung epithelial cells, ATCC) and HeLa cells (human cervix adenocarcinoma epithelial cells, ATCC) were maintained in Kaighn’s modified Ham F-12 nutrient mixture medium with 10% FBS and penicillin-streptomycin. All cells were cultured and maintained at 37°C with 5% CO_2_. For PB1 ectopic expression, lentiviral vectors were co-transfected with an expression plasmid into HEK293 cells. The medium was changed the following day and the virus-containing supernatant was collected at 48 and 72 hours (h) after transfection, filtered through a 0.22-μm filter, and subsequently used to infect A549 cells with Polybrene (8 mg/ml). Transduced cells were purified by puromycin selection. For PB1 knockdown cells, target sequences (*PB1*-targeting shRNA sequence: GCTATGGATTCGTGGCTAA) were cloned into pSUPER digested with Bgl II/Hind III. Transfected A549 cells were purified by puromycin selection. For *NBR1*^*-/-*^ and *RNF5*^*-/-*^ cells, target sequences (*NBR1*-targeting sgRNA sequence: GTTGGGCTGATATCGAAGCTA; *RNF5*-targeting sgRNA sequence: GCGCTCGCGATTTGGCCCTTC) were cloned into pGK1.1 digested with BpiI. Transfected HEK293 cells were purified by puromycin selection. *NDP52*^*-/-*^ cells were kindly provided by Prof. Jun Cui (Sun Yat-sen University, China). *MAVS*^*-/-*^ cells were kindly provided by Prof. Xin Cao (Jilin Agricultural University, China). *ATG7*^*-/-*^ cells were kindly provided by Prof. Chan Ding (Shanghai Veterinary Research Institute, Chinese Academy of Agricultural Sciences, China).

A/Environment/Suzhou/SZ19/2014(H7N9) (SZ19) was isolated and stored in our laboratory. SZ19-ΔF2 and SZ19-ΔF2-mut viruses were rescued by use of reverse genetics. Recombinant vesicular stomatitis virus expressing green fluorescent protein (VSV-GFP) was generated as described previously [73]. Sendai virus (SeV) was kindly provided by Prof. Hongkui Deng (Peking University, China). Viruses were inoculated into and propagated in 9-day-old specific-pathogen-free (SPF) chicken embryos for 3 days and then harvested and stored at -80°C until use.

The genes encoding viral proteins were amplified from the SZ19 genome and cloned into the pRK expression vector with Flag, HA, Myc, or GFP tags at the N-terminus by using standard molecular biology techniques. Plasmids for HA-, Flag-, and Myc-tagged RIG-I, RIG-IN, MDA5-N, MAVS, TRAF3, TBK1, IKKε, IRF3, and ubiquitin (HA-ubiquitin or its mutants)[74], and use of the IFN-β-Luc, ISRE-Luc, NF-κB-Luc, STAT1-Luc, and pRL-TK internal control luciferase reporter plasmids used in the study were described previously [35]. Plasmids for Flag/Myc/GFP-tagged NBR1, OPTN, p62, NDP52, Tollip, RNF5, MARCH8, USP25, and GAPDH were kindly provided by Dr. Yu Zhang (Huazhong University of Science and Technology, China). Plasmids for GFP-LC3 and cherry-MAVS were kindly provided by Prof. Hong-Bing Shu (Wuhan University, China). The plasmid for pttRFP-GFP-LC3 was kindly provided by Prof. Chan Ding (Shanghai Veterinary Research Institute, Chinese Academy of Agricultural Sciences, China). Flag-tagged PB1 from H1N1, H5N1, and H9N2 viruses, and Flag-PB1ΔF2, Flag-PB1-ΔF2-mut, Flag-GFP, MAVS truncations, NBR1 truncations, and MAVS mutants (K7R, K10R, K136R, K362R, K461R, and K362, 461R) were constructed and cloned into pRK by using standard molecular biology techniques. His-tagged MAVS, PB1, NP, and NBR1 were constructed and cloned into the pET-28a prokaryotic expression vector, and GST-tagged PB1, NBR1, and RNF5 were constructed and cloned into the pGEX-4T-1 prokaryotic expression vector by using standard molecular biology techniques.

### Antibodies and reagents

The antibodies used in this study were as follows: anti-GFP (11814460001), HRP-conjugated anti-HA (12013819001), Myc (11814150001) antibodies (Roche); phosphorylated TBK1(5483), IκBα (9246), IRF3 (4961) antibodies (Cell Signaling Technology); anti-GST-Tag (M20007L) and HRP-conjugated goat anti-mouse secondary antibody (M21001L) (Abmart); anti-His (TA-02), HRP-conjugated goat anti-rabbit IgG (ZB-2301), Alexa Fluor 488-conjugated anti-mouse IgG (ZF-0512), Alexa Fluor 594-conjugated goat anti-rabbit IgG (ZF-0516) secondary antibodies (ZSGB-BIO); anti-NBR1 (16004-1-AP), anti-TRAF3 (18099-1-AP) and anti-NDP52 (12229-1-AP) antibodies (Proteinteck); anti-MAVS antibody (BETHYL, A300-782A); anti-RNF5 (ab128200), anti-GAPDH (ab181602), anti-ubiquitin (K63) (ab179434), anti-ubiquitin(K48) (ab140601), anti-ubiquitin(K27) (ab181537) antibodies (Abcam); anti-ubiquitin (WT) antibody (Santa Cruz, sc-8017); anti-LC3(PM036) and anti-ATG7 (PM039) antibodies (MBL); anti-LAMP1 (11215-R107) and anti-NP (11675-MM03T) antibodies (Sino Biological); anti-p62/SQSTM1 (P0067) and HRP-conjugated anti-Flag (A8592) antibodies (Sigma); Cy3-labeled goat anti-mouse IgG (A0521), Dylight 405-conjugated goat anti-rabbit IgG (A0605) and Dylight 405-conjugated goat anti-mouse IgG (A0609) secondary antibodies (Beyotime); and mouse anti-PB1 monoclonal antibody (prepared in our laboratory). Reagents used in the study included: poly(dA:dT) (tlrl-patc) and poly(I:C) (tlrl-picwlv) (InvivoGen); a human IFN-β DuoSet ELISA kit (R&D, DY814-05); 3-Methyladenine (M9281), MG132 (M7449), DMSO (D2650), CQ (Chloroquine phosphate) (PHR1258), NH_4_Cl (09718), Earle’s balanced salt solution (EBSS), anti-Flag agarose affinity beads (A2220) and protein A/G agarose affinity beads (P6486/E3403) (Sigma); Glutathione Sepharose 4B (GE Healthcare, 17-0756-05); Bafilomycin A1(Baf-A1) (Selleck, S1314); DAPI (C1005), ZVAD (Caspase inhibitor Z-VAD-FMK, C1202), NP-40 (ST366), and recombinant Human IFN-β (P5660) (Beyotime). Human RNF5, ULK1, ATG13, FIP200, and ATG101 specific siRNAs were designed and synthesized by RiboBio Co. Ltd, China.

### Luciferase reporter assay

HEK293 cells (1×10^5^) grown in 24-well plates were co-transfected with Luciferase reporter plasmids (IFN-β-Luc, ISRE-Luc, NF-κB-Luc, or STAT1-Luc) and pRL-TK plasmid, along with the indicated amount of control plasmid or plasmids expressing PB1 or other molecules. At 24 h post-transfection, the cells were left untreated or were treated with poly(dA:dT), poly(I:C), IFN-β, SeV, SZ19-ΔF2, or SZ19-ΔF2-mut for another 12 h. Cell lysates were prepared and measured for firefly and Renilla luciferase activities by using the Dual-Luciferase Reporter Assay System, following the manufacturer’s instructions. All reporter assays were repeated at least three times.

### RNA isolation and quantitative RT-PCR

Total RNAs from HEK293 cells or PB1-expressing A549 cells transfected with the indicated molecules stimulated with or without SZ19 or SeV were extracted by use of TRIzol (Invitrogen, CA, USA) following the manufacturer’s instructions and were subsequently transcribed into cDNA by use of Moloney murine leukemia virus (M-MLV) reverse transcriptase (Promega, WI, USA) according to the manufacturer’s protocol. Quantitative PCR (qPCR) was performed using a FastSYBR mixture on a real-time PCR system (Applied Biosystems 7500, CA, USA). Gene-specific primers for human genes were synthesized; primer sequences are shown in S1 Table. Gene expression levels were normalized to that for glyceraldehyde-3-phosphate dehydrogenase (*GAPDH*) and are presented as changes (n-fold) in induction relative to the control value. All assays were repeated at least three times.

### Coimmunoprecipitation

HEK293 cells or A549 cells were co-transfected with the indicated molecules with or without virus infection for 24 h. The transfected cells were then harvested and lysed in NP-40 lysis buffer (20 mM Tris-HCl (pH 7.5), 150 mM NaCl, 1% NP-40, 1 mM EDTA with protease inhibitor cocktails). For each immunoprecipitation, 1 ml of lysate was incubated for 4 h at 4°C with 0.5 μg of the indicated antibody or control IgG and 30 μL of protein A/G-Sepharose in 20% ethanol (SIGMA). The beads were washed three times with 1 ml of lysis buffer containing 500 mM NaCl. The precipitates were analyzed by using standard immunoblotting procedures. The intensities of the target bands were quantified by using the Image J program (NIH, USA).

### VSV-GFP bioassay

Antiviral cytokine secretion bioassays were conducted as previously described, with slight modifications [75]. Briefly, HEK293 cells were grown in 24-well plates and transfected with the indicated plasmids. At 24 h post-transfection, the cells were infected with SeV (MOI = 1) for another 24 h. The supernatants were harvested and inactivated by placing the samples on ice with a 30-W ultraviolet radiation lamp for 20 min. The ultraviolet radiation -inactivated supernatant was added to fresh confluent cells and incubated for 24 h. The cells were then infected at an MOI of 0.01 with VSV-GFP. At 12 h post-infection, VSV-GFP replication was visualized by monitoring the GFP expression level by fluorescence microscopy or flow cytometry.

### GST pull-down assay

GST pull-down assays were conducted as previously described with slight modifications [55], Briefly, the encoded GST- or His-tagged fusion proteins and the control GST proteins were expressed in BL21 cells after induction with 0.1 mmol/L IPTG overnight at 18°C. Centrifuged cells were resuspended in lysis buffer (1 × PBS, 0.2 mM PMSF, 1% Triton X-100) and sonicated for 15 min. After centrifugation, the supernatant was applied to a Glutathione–Sepharose 4B bead column (GE Healthcare Bio-Sciences) or ProteinIso Ni-NTA Resin (TransGen Biotech, China), following the manufacturer’s instructions. Purified GST/His-tagged fusion proteins were diluted with 1× PBS and filtered through Amicon Ultra 0.5 ml filters (Millipore). Then, 1 μg of purified GST protein or GST fusion protein (GST–PB1) was captured by Glutathione–Sepharose 4B beads (GE Healthcare) for the GST pull-down assays, and His-tagged fusion protein was added overnight at 4°C. The beads were then washed three times with ice-cold PBS. The supernatant was loaded onto gels, followed by immunoblotting with anti-His antibody.

### Confocal microscopy

Confocal microscopy was performed as previously described [55]. Cells were seeded in 12-well plates (5 × 10^5^ cells/well) on coverslips. Twenty-four hours after transfection, the cells were fixed with 4% paraformaldehyde for 20 min at room temperature, and then washed three times with PBS. Cells were permeabilized with 0.1% Triton X-100 in PBS for 10 min and blocked with 5% skimmed milk for 1 h. Then, the cells were incubated with the indicated primary and secondary antibodies and DAPI. The stained cells were observed with a Leica microscope (TCS SP8) with a 100× oil objective. The fluorescence intensity profile of the indicated proteins was measured by using the Image J program.

### Detection of ubiquitin-modified proteins

The experiments were performed as previously described [76]. Briefly, the cells were lysed in lysis buffer containing 1% SDS and denatured by heating at 95°C for 10 min. After centrifugation, the supernatants were diluted with NP-40 lysis buffer until the concentration of SDS was 0.1%, and were then coimmunoprecipitated with the indicated antibodies. Ubiquitin-modified proteins were detected by immunoblotting with the indicated antibodies.

### Virus titration

Virus titers of virus-containing culture supernatant were determined by end-point titration in eggs. For end-point viral titration in eggs, 10-fold serial dilutions of each sample were inoculated into 9-day-old SPF eggs. Sixty hours after inoculation, fluid from the allantoic cavity was collected and tested for the ability to agglutinate chicken erythrocytes as an indicator of viral replication. Infectious virus titers are reported as log_10_ EID_50_/ml, and were calculated from three replicates by using the method of Reed-Muench [77].

### Statistical analysis

Data are presented as the mean ± SD unless otherwise indicated. Student’s t test was used for all statistical analyses with the GraphPad Prism 5 software (GraphPad Software, San Diego, CA, USA). Differences between groups were considered significant when the *P* value was < 0.05(*), < 0.01(**), < 0.001(***), and < 0.0001(****).

## Supporting information

S1 Fig H7N9 PB1 inhibits type I IFN signaling pathway. (A) PB1-F2 is silenced in the PB1 ORF. The upper panel shows the nucleotide acid and amino acid sequences of the PB1 (red) and PB1-F2 (blue) ORF in the PB1 ORF. The italicized nucleotide acids were mutated to the green-labeled nucleotide acids in the recombinant plasmids and virus. The lower panel shows the sequence of the recombinant plasmids. (B) Screening of viral proteins of SZ19 virus for their effects on SeV- and poly(I:C)-induced activation of IFN-β promoter. HEK293 cells were transfected with IFN-β promoter and plasmids encoding viral proteins (PB2, PB1, PA, HA, NP, NA, M1, M2, NS1, NS2, PB1-F2 and PAX) for 24 h. The cells were then infected with SeV or transfected with poly(I:C) for 12 h before luciferase analysis. **(C)** PB1 inhibits SeV- and poly(I:C)-induced transcription of *IFN-β* and downstream genes. HEK293 cells were transfected with PB1, NS1 and GFP for 24 h. The cells were then infected with SeV or transfected with poly(I:C) for 12 h before qPCR analysis were performed. (D) PB1 from different subtypes of influenza A virus inhibits SeV-induced transcription of *IFN-β* and downstream genes. A549 cells were transfected with PB1 from different subtypes of influenza A virus and NP from SZ19 virus for 24 h. The cells were then infected with SeV for 12 h before qPCR analysis was performed. The data shown represent three independent experiments; bars represent the mean ± SD of the three independent experiments (n = 3). [*P*< 0.05(*), *P* < 0.01(**), *P* < 0.001(***), *P* < 0.0001(****)].
(TIF)

S2 Fig H7N9 PB1 promotes degradation of MAVS through the autophagic pathway. (A) Schematic representation of the domain organization of MAVS and its interaction with PB1. -, no interaction; +, interaction. (B) PB1 from different subtypes of influenza A virus decreases the MAVS protein level. HEK293 cells were transfected with Myc-MAVS and Flag-PB1 from different subtypes of influenza A virus and NP from SZ19 virus for 24 h before immunoblot analysis. (C) HeLa cells were transfected with pRFP-GFP-LC3 and an empty vector or Flag-PB1. At 24 h post-transfection, cells were treated with EBSS or left untreated for the indicated times and then analyzed for autophagosome formation. The data shown represent three independent experiments.
(TIF)

S3 Fig PB1 enhances NBR1-mediated degradation of MAVS. (A) Knockdown of ULK1, ATG13, FIP200, and ATG101 has no marked effect on PB1-mediated MAVS degradation. HEK293 cells were transfected with siRNA for NC, ULK1, ATG13, FIP200, and ATG101 (100 nM/well) for 24 h, the cells then were transfected with indicated plasmids for another 24 h before immunoblot analysis with the indicated antibodies (upper panels). The lower chart shows the efficiency of siRNA for ULK1, ATG13, FIP200, and ATG101. HEK293 cells were transfected with siRNA for control, ULK1, ATG13, FIP200, and ATG101 (100 nM/well) for 24 h before qPCR analysis. (B) MAVS interacts with NBR1, OPTN, p62, and NDP52. HEK293 cells were transfected with the indicated plasmids for 24 h before co-immunoprecipitation and immunoblotting analyses with the indicated antibodies. (C) Schematic representation of the domain organization of NBR1 and its interaction with MAVS. -, no interaction; +, interaction. The data shown represent three independent experiments; bars represent the mean ± SD of the three independent experiments (n = 3). [*P*< 0.05(*), *P* < 0.01(**), *P* < 0.001(***), *P* < 0.0001(****)].
(TIF)

S4 Fig The identification of the key lysine residue for H7N9 PB1-mediated degradation of MAVS. (A) HEK293 cells were transfected with Myc-MAVS and the indicated mutants in the presence or absence of Flag-PB1 for 24 h before immunoblot analysis. (B) The effects of MAVS-WT and MAVS-K362/461R on SZ19-ΔF2 virus replication. Wild-type and *MAVS*^-/-^ HEK293 cells were transfected with MAVS-WT or MAVS-K362/461R plasmid for 24 h. The cells were then infected with SZ19-ΔF2 virus (MOI = 0.01) for another 48 h. The supernatants were harvested for virus titration (EID_50_/ml). The data shown represent three independent experiments; bars represent the mean ± SD of the three independent experiments (n = 3). [*P*< 0.05(*), *P* < 0.01(**), *P* < 0.001(***), *P* < 0.0001(****); ‘ns’ indicates no significant difference].
(TIF)

S1 Table PCR primers used in this study. (XLSX)
