# Supplementary figures and images for: The PB1 protein of influenza A virus inhibits the innate immune response by targeting MAVS for NBR1-mediated selective autophagic degradation
Source: PLoS Pathog. 2021 Feb 12;17(2):e1009300. doi: 10.1371/journal.ppat.1009300 (PMC7880438; doi:10.1371/journal.ppat.1009300)

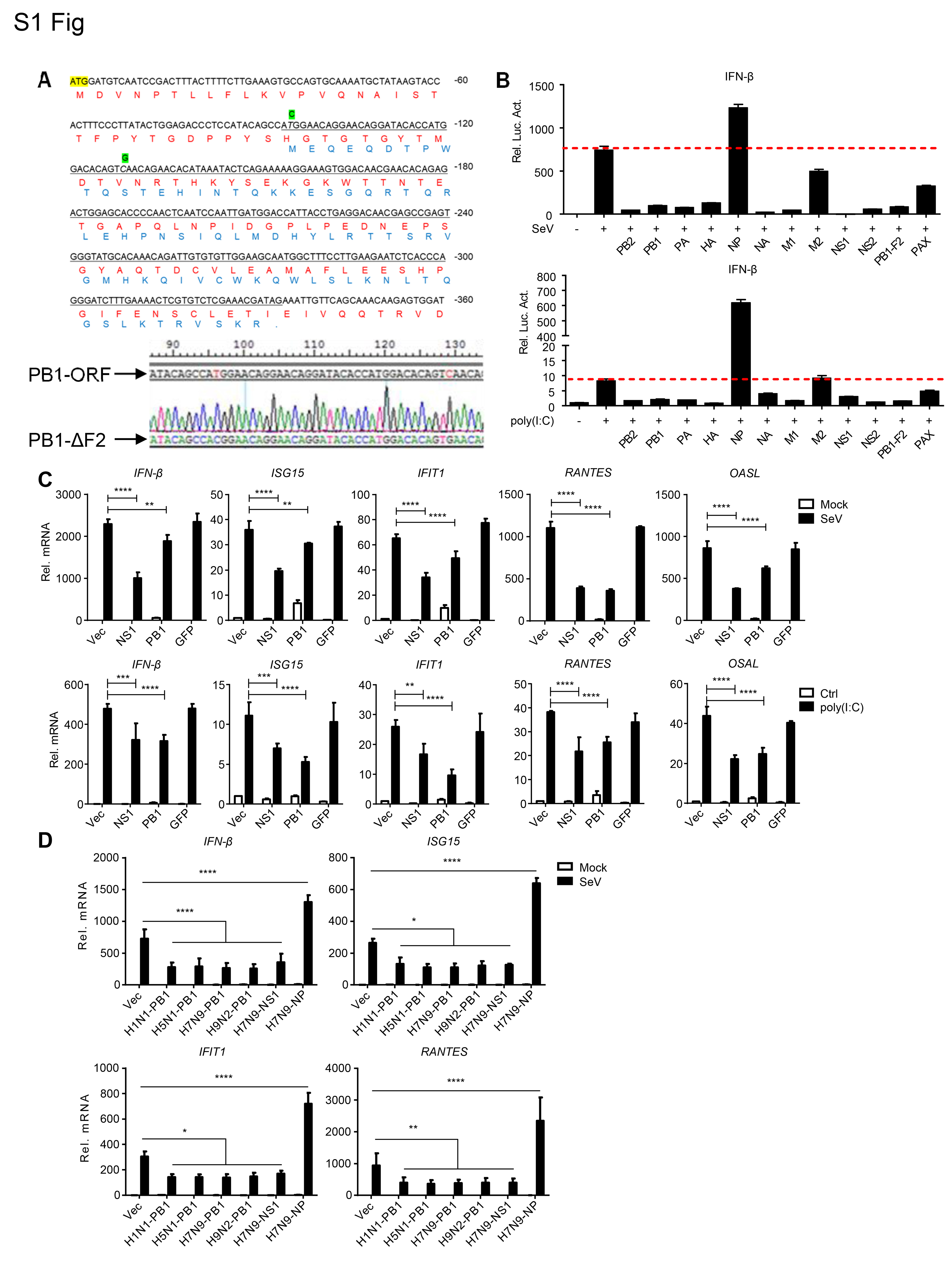

Supplement: S1 Fig — (A) PB1-F2 is silenced in the PB1 ORF. The upper panel shows the nucleotide acid and amino acid sequences of the PB1 (red) and PB1-F2 (blue) ORF in the PB1 ORF. The italicized nucleotide acids were mutated to the green-labeled nucleotide acids in the recombinant plasmids and virus. The lower panel shows the sequence of the recombinant plasmids. (B) Screening of viral proteins of SZ19 virus for their effects on SeV- and poly(I:C)-induced activation of IFN-β promoter. HEK293 cells were transfected with IFN-β promoter and plasmids encoding viral proteins (PB2, PB1, PA, HA, NP, NA, M1, M2, NS1, NS2, PB1-F2 and PAX) for 24 h. The cells were then infected with SeV or transfected with poly(I:C) for 12 h before luciferase analysis. (C) PB1 inhibits SeV- and poly(I:C)-induced transcription of IFN-β and downstream genes. HEK293 cells were transfected with PB1, NS1 and GFP for 24 h. The cells were then infected with SeV or transfected with poly(I:C) for 12 h before qPCR analysis were performed. (D) PB1 from different subtypes of influenza A virus inhibits SeV-induced transcription of IFN-β and downstream genes. A549 cells were transfected with PB1 from different subtypes of influenza A virus and NP from SZ19 virus for 24 h. The cells were then infected with SeV for 12 h before qPCR analysis was performed. The data shown represent three independent experiments; bars represent the mean ± SD of the three independent experiments (n = 3). [P< 0.05(*), P < 0.01(**), P < 0.001(***), P < 0.0001(****)]. (TIF) [file ppat.1009300.s001.tif]

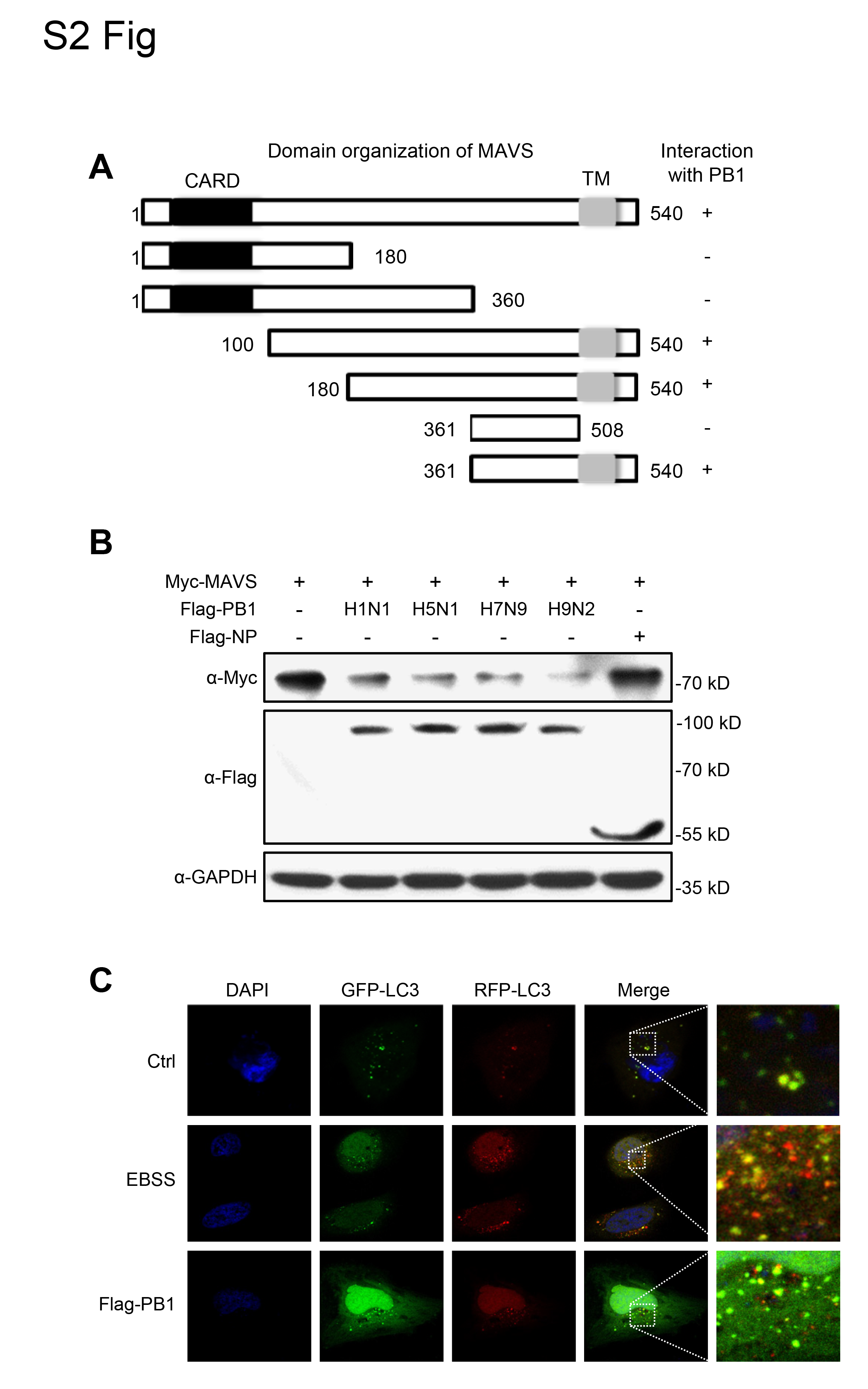

Supplement: S2 Fig — (A) Schematic representation of the domain organization of MAVS and its interaction with PB1. -, no interaction; +, interaction. (B) PB1 from different subtypes of influenza A virus decreases the MAVS protein level. HEK293 cells were transfected with Myc-MAVS and Flag-PB1 from different subtypes of influenza A virus and NP from SZ19 virus for 24 h before immunoblot analysis. (C) HeLa cells were transfected with pRFP-GFP-LC3 and an empty vector or Flag-PB1. At 24 h post-transfection, cells were treated with EBSS or left untreated for the indicated times and then analyzed for autophagosome formation. The data shown represent three independent experiments. (TIF) [file ppat.1009300.s002.tif]

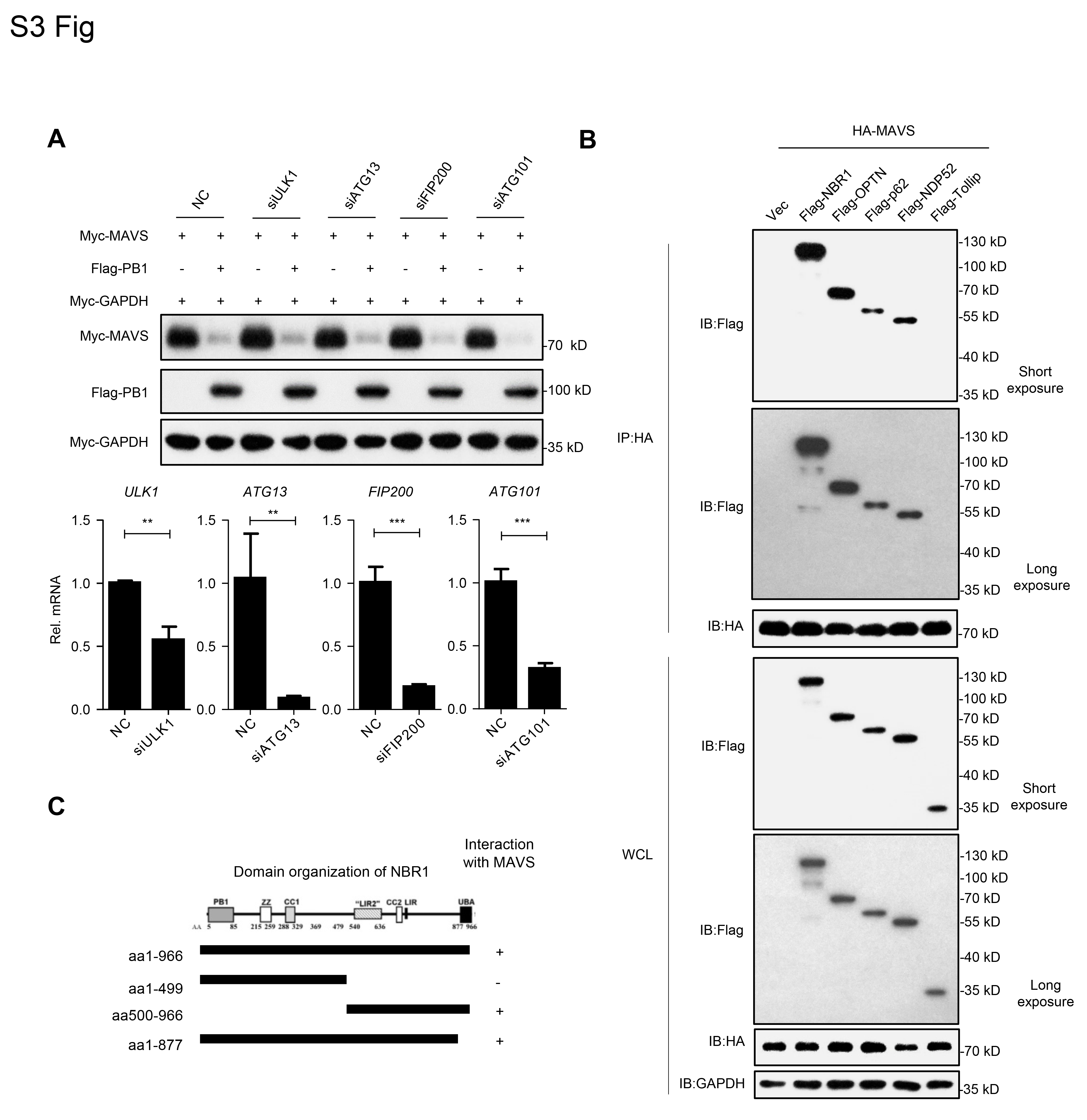

Supplement: S3 Fig — (A) Knockdown of ULK1, ATG13, FIP200, and ATG101 has no marked effect on PB1-mediated MAVS degradation. HEK293 cells were transfected with siRNA for NC, ULK1, ATG13, FIP200, and ATG101 (100 nM/well) for 24 h, the cells then were transfected with indicated plasmids for another 24 h before immunoblot analysis with the indicated antibodies (upper panels). The lower chart shows the efficiency of siRNA for ULK1, ATG13, FIP200, and ATG101. HEK293 cells were transfected with siRNA for control, ULK1, ATG13, FIP200, and ATG101 (100 nM/well) for 24 h before qPCR analysis. (B) MAVS interacts with NBR1, OPTN, p62, and NDP52. HEK293 cells were transfected with the indicated plasmids for 24 h before co-immunoprecipitation and immunoblotting analyses with the indicated antibodies. (C) Schematic representation of the domain organization of NBR1 and its interaction with MAVS. -, no interaction; +, interaction. The data shown represent three independent experiments; bars represent the mean ± SD of the three independent experiments (n = 3). [P< 0.05(*), P < 0.01(**), P < 0.001(***), P < 0.0001(****)]. (TIF) [file ppat.1009300.s003.tif]

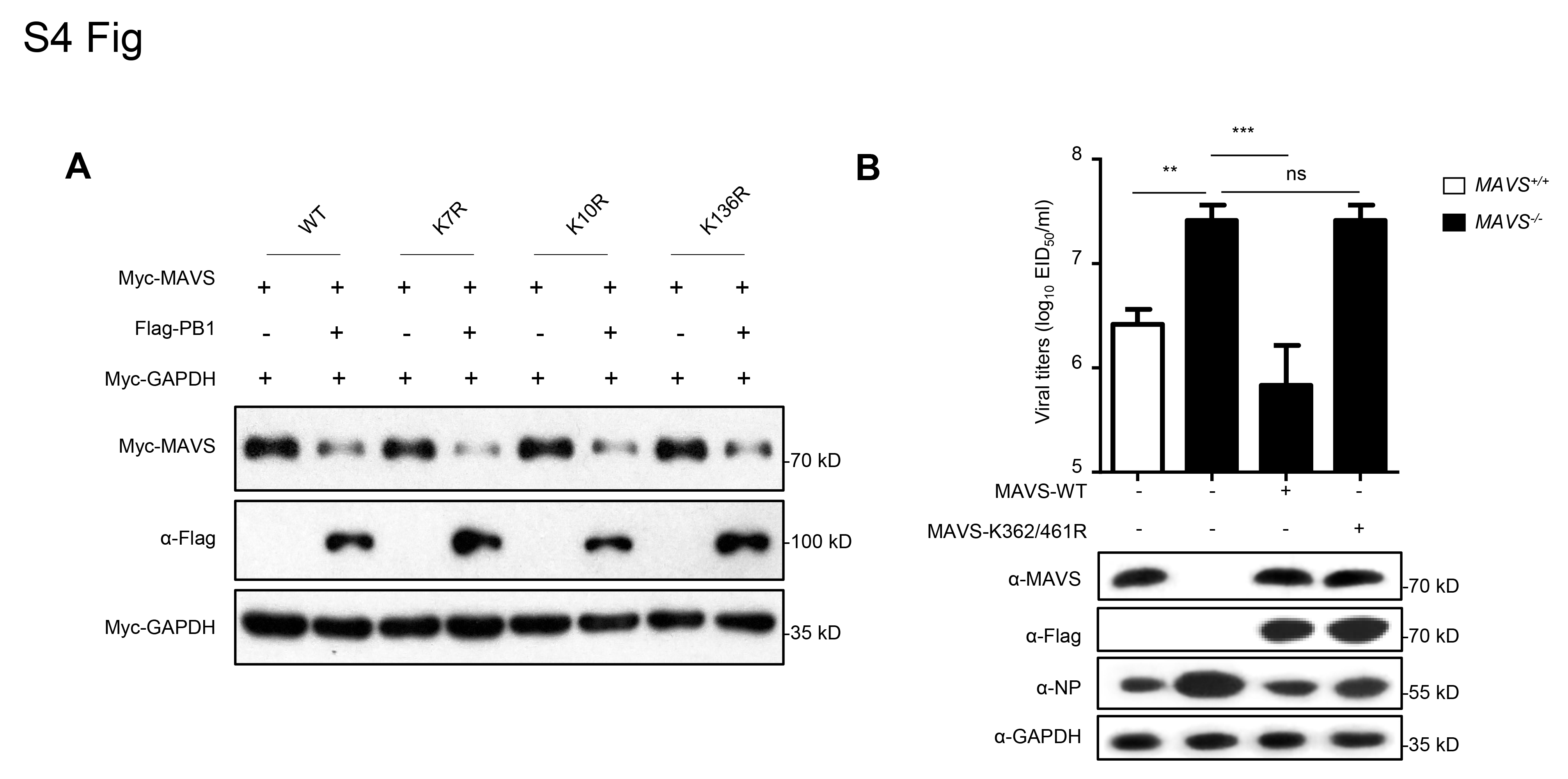

Supplement: S4 Fig — (A) HEK293 cells were transfected with Myc-MAVS and the indicated mutants in the presence or absence of Flag-PB1 for 24 h before immunoblot analysis. (B) The effects of MAVS-WT and MAVS-K362/461R on SZ19-ΔF2 virus replication. Wild-type and MAVS-/- HEK293 cells were transfected with MAVS-WT or MAVS-K362/461R plasmid for 24 h. The cells were then infected with SZ19-ΔF2 virus (MOI = 0.01) for another 48 h. The supernatants were harvested for virus titration (EID50/ml). The data shown represent three independent experiments; bars represent the mean ± SD of the three independent experiments (n = 3). [P< 0.05(*), P < 0.01(**), P < 0.001(***), P < 0.0001(****); ‘ns’ indicates no significant difference]. (TIF) [file ppat.1009300.s004.tif]
